# Genetic mapping of APP and amyloid-β biology modulation by trisomy 21

**DOI:** 10.1523/JNEUROSCI.0521-22.2022

**Published:** 2022-07-14

**Authors:** Paige Mumford, Justin Tosh, Silvia Anderle, Eleni Gkanatsiou Wikberg, Gloria Lau, Sue Noy, Karen Cleverley, Takashi Saito, Takaomi C. Saido, Eugene Yu, Gunnar Brinkmalm, Erik Portelius, Kaj Blennow, Henrik Zetterberg, Victor Tybulewicz, Elizabeth M.C. Fisher, Frances K. Wiseman

**Affiliations:** 1 The UK Dementia Research Institute, University College London, Queen Square, London WC1N 3BG, UK; 2 Department of Neuromuscular Diseases, Queen Square Institute of Neurology, University College London, Queen Square, London WC1N 3BG, UK; 3 Department of Psychiatry and Neurochemistry, Institute of Neuroscience and Physiology, University of Gothenburg, S-431 80, Sweden; 4 Laboratory for Proteolytic Neuroscience, RIKEN Brain Science Institute, Wako-shi, Saitama, Japan; 5 The Children’s Guild Foundation Down Syndrome Research Program, Genetics and Genomics Program and Department of Cancer Genetics and Genomics, Roswell Park Comprehensive Cancer Center, Buffalo, NY, USA; 6 Clinical Neurochemistry Laboratory, Sahlgrenska University Hospital, S-431 80 Mölndal, Sweden; 7 Department of Neurodegenerative Disease, UCL Institute of Neurology, London WC1N 3BG, UK; 8 Hong Kong Center for Neurodegenerative Diseases, Hong Kong, China; 9 The Francis Crick Institute, London NW1 1AT, UK; 10 Department of Immunology and Inflammation, Imperial College, London W12 0NN, UK

**Keywords:** Amyloid precursor protein (APP), amyloid-β, Down syndrome, DYRK1A, BACE2

## Abstract

Individuals who have Down syndrome (DS) frequently develop early onset Alzheimer’s disease (AD), a neurodegenerative condition caused by the build-up of aggregated amyloid-β and tau proteins in the brain. Amyloid-β is produced by amyloid precursor protein *(APP)*, a gene located on chromosome 21. People who have Down syndrome have three copies of chromosome 21 and thus also an additional copy of *APP*; this genetic change drives the early development of Alzheimer’s disease in these individuals. Here we use a combination of next-generation mouse models of Down syndrome (Tc1, Dp3Tyb, Dp(10)2Yey and Dp(17)3Yey) and a knockin mouse model of amyloid-β accumulation (*App^NL-F^*) to determine how chromosome 21 genes, other than *APP*, modulate APP/amyloid-β in the brain when in three copies. Using both male and female mice, we demonstrate that three copies of other chromosome 21 genes are sufficient to partially ameliorate amyloid-β accumulation in the brain. We go on to identify a subregion of chromosome 21 that contains the gene/genes causing this decrease in amyloid-β accumulation and investigate the role of two lead candidate genes *Dyrk1a* and *Bace2*. Thus an additional copy of chromosome 21 genes, other than *APP*, can modulate APP/amyloid-β in the brain under physiological conditions. This work provides critical mechanistic insight into the development of disease and an explanation for the typically later age of onset of dementia in people who have ADDS, compared to those who have familial AD caused by triplication of *APP*.

## Introduction

DS is caused by trisomy of human chromosome 21 (Hsa21), and occurs in around 1/1000 live births in Europe (de Graaf, Buckley et al. 2021). Most individuals who have DS develop the neuropathological features of AD; amyloid-β plaques and tau neurofibrillary tangles by the age of 50 (Davidson, Robinson et al. 2018), and 80% of individuals will have developed dementia by age 65 (McCarron, McCallion et al. 2017). The high prevalence of AD in DS is in part due to the gene encoding amyloid precursor protein *(APP)* being located on Hsa21, thereby raising APP and amyloid-β protein levels (Glenner and Wong 1984, Cheon, Dierssen et al. 2008, Doran, Keator et al. 2017). Recent studies in preclinical systems have demonstrated that an extra copy of other genes on Hsa21 can modulate APP biology (Garcia-Cerro, Rueda et al. 2017, Wiseman, Pulford et al. 2018, Alic, Goh et al. 2020, Tosh, Rhymes et al. 2021) and thus may alter the earliest stages of AD in individuals who have DS. These extra genes may act to *promote* or to *reduce* amyloid-β accumulation, the mechanism that predominates is currently unclear. Notably, the age of clinical dementia diagnosis occurs slightly later in individuals who have DS, compared with those who have early-onset familial AD caused by duplication of *APP* (*i.e*., with three copies of wild-type *APP)* (Wiseman, Al-Janabi et al. 2015). However, a direct comparison between these two causes of AD is confounded by the different diagnostic criteria used (as necessitated by the underlying intellectual disability that occurs in people who have DS) (Benejam, Videla et al. 2020). Understanding these processes is crucial to the appropriate selection of treatments for AD-primary prevention trials in people who have DS.

Previous *in vivo* studies have either examined the processing of endogenous mouse APP or used *APP* transgenic models to address this biology, but both of these approaches have limitations (Garcia-Cerro, Rueda et al. 2017, Sasaguri, Nilsson et al. 2017, Wiseman, Pulford et al. 2018, Tosh, Rhymes et al. 2021). Mouse APP differs in sequence from the human protein. In the amyloid-β region these differences reduce both cleavage of the protein by β-secretase and the tendency of the amyloid-β generated to aggregate (Serneels, T’Syen et al. 2020), thus limiting our ability to determine how changes to biology affect accumulation of amyloid-β – a key early aspect of AD. The over- and mis-expression of *APP* in transgenic mouse models may cause artefactual phenotypes, masking the modulatory effect of the extra copy of Hsa21 genes and causing elevated mortality which may confound data interpretation (Saito, Matsuba et al. 2014, Sasaguri, Nilsson et al. 2017).

**Figure 1:**
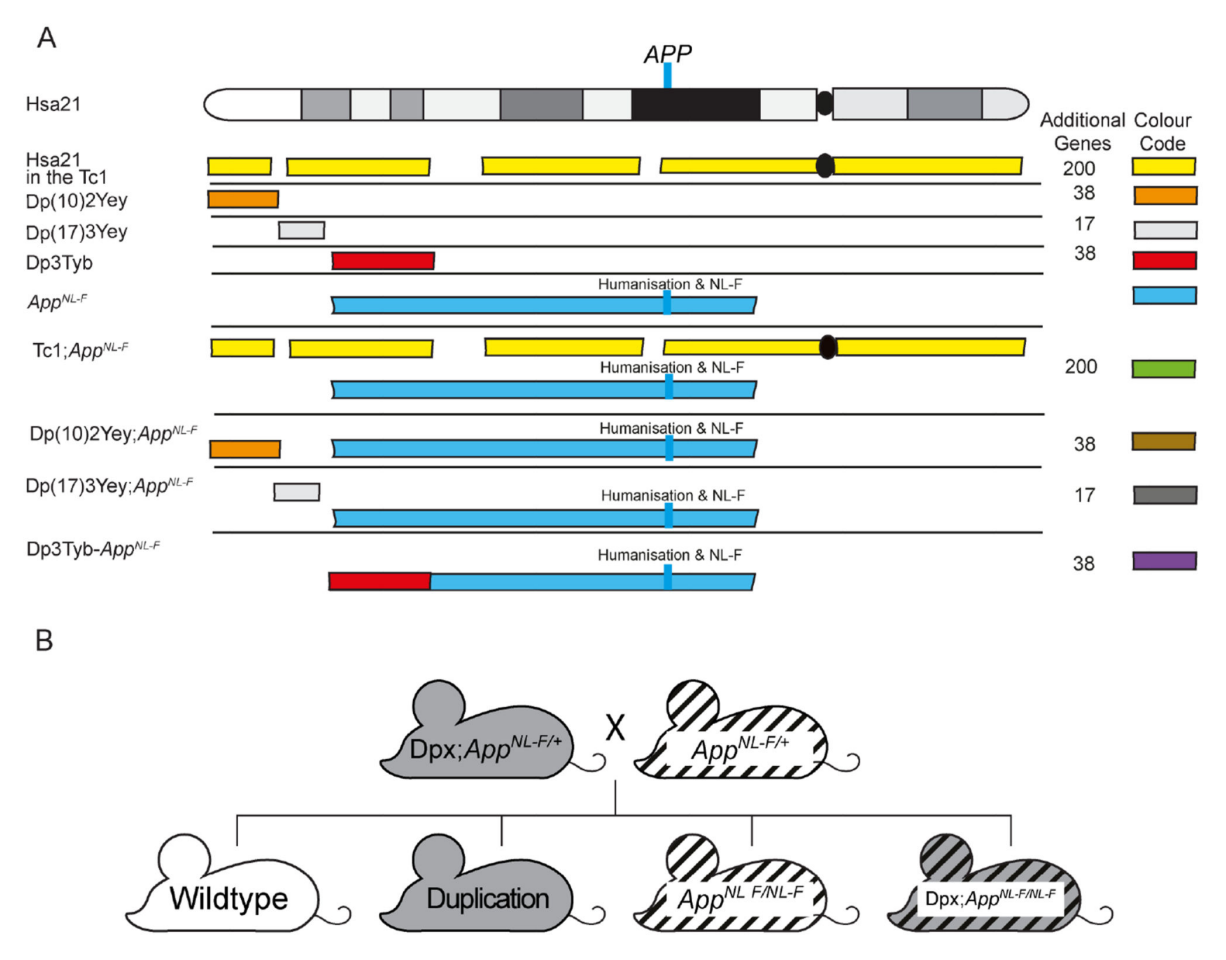
A schematic of Hsa21 indicating the major karyotypic bands, and the regions of Hsa21 or homologous regions of mouse chromosomes that are in three-copies in the mouse models used. **(A)** The additional Hsa21 gene content in Tc1 (yellow), in the Dp(10)2Yey (orange), Dp(17)3Yey (light grey) and Dp3Tyb (red) segmental duplication models of DS as shown. The number of additional human genes in the Tc1, and the additional number of mouse genes in the Dp(10)2Yey, Dp(17)3Yey and Dp3Tyb as listed. These lines were crossed with the *App^NL-F^* mouse model of amyloid-β accumulation (blue), to generate *Tc1;App^NL-F^*(green), Dp(10)2Yey;*App^NL-F^* (brown), Dp(17)3Yey;*App^NL-F^*(dark grey), and Dp3Tyb-*App^NL-F^* (purple) offspring, homozygous for the *App^NL-F^* allele and carrying the additional gene content. The location of *APP/App* is indicated by the blue line, this locus is altered in the *App^NL-F^* model such that the amyloid-β sequence is humanised and the model carries the AD-causal Swedish (NL) and Iberian (F) mutations. This colour scheme is used in subsequent figures to code for each of the mouse models. (**B)** Schematic of the generation of experimental cohorts for the duplication models (Dp(10)2Yey, Dp(17)3Yey and Dp3Tyb) used in the studies, experimental cohorts were produced by crossing *Dpx;App^NL-F/+^* mice with *App^NL-F/+^* mice.

Here we take a combinatorial approach: assessing the effect of an additional copy of Hsa21 genes (using a series of DS mouse models (O’Doherty, Ruf et al. 2005, Yu, Li et al. 2010, Lana-Elola, Watson-Scales et al. 2016)) on the biology of endogenous mouse APP and on APP generated from a partially humanised *App* knock-in allele that also carries AD-causal Swedish (NL) and Iberian (F) point mutations, which does not cause elevated mortality (*App^NL-F^)* (Fig. 1) (Saito, Matsuba et al. 2014). These data indicate that trisomy of genes on Hsa21 reduces amyloid-β accumulation and that people who have DS are partly protected from their raised *APP* gene dose by the additional copy of other genes on the chromosome. We go on to show that one of the gene or genes that cause this change in biology is located on mouse chromosome 16 between *Mir802* and *Zbtb21*. This region contains 38 genes and we specifically test if mechanisms linked to two lead candidate genes in this region, *Dyrk1a* and *Bace2*, occur in our novel *in vivo* AD-DS model system.

## Methods

### Animal welfare and husbandry

All experiments were undertaken in accordance with the Animals (Scientific Procedures) Act 1986 (United Kingdom), after local institutional ethical review by the Medical Research Council, University College London and in accordance with ARRIVE2 guidelines (Percie du Sert, Hurst et al. 2020). Mice were housed in individually ventilated cages (Techniplast) with grade 5, autoclaved dust-free wood bedding, paper bedding and a translucent red “mouse house” at a specific pathogen free facility. Free-access to food (Picolab Rodent Diet 20 Labdiet) and water was provided. The animal facility was maintained at a constant temperature of 19-23°C with 55 ± 10% humidity in a 12 h light/dark cycle. Pups were weaned at 21 days and moved to standardised same-sex group housing with a maximum of 5 mice per cage.

The following mouse strains were used in this paper, here we show abbreviated name and then the official name and unique Mouse Genome Informatics (MGI) identifier: *App^NL-F^* (App^tm2.1Tcs^, MGI:5637816), Tc1 (Tc(HSA21)1TybEmcf, MGI:3814712), Dp3Tyb (Dp(16Mir802-Zbtb21)3TybEmcf, MGI:5703802), Dp(10)2Yey (Dp(10Prmt2-Pdxk)2Yey, MGI:4461400) and Dp(17)3Yey (Dp(17Abcg1-Rrp1b)3Yey, MGI:4461398).

Tc1 mice were maintained by mating Tc1 females to F1 (129S8 × C57BL/6) males. All other mouse strains were maintained by backcrossing males and females to C57BL/6J mice (imported from the Jackson Laboratory). Experimental cohorts for Tc1, Dp(10)2Yey and Dp(17)3Yey studies were produced by crossing mice carrying the additional Hsa21 or Hsa21 orthologous duplications with *App^NL-F/+^* animals in a two-generation cross to generate all required genotypes from the second generation (wild-type, Tc1/Dpx, *App^NL-F/NL-F^*, Tc1/Dpx;*App^NL-F/NL-F^*, Fig. 1, Table 1). As both the Dp3Tyb segmental duplication and the *App^NL-F^* gene are located on mouse chromosome 16 (Mmu16), for this cross we first generated a Dp3Tyb*-App^NL-F^* recombinant Mmu16, by crossing the two lines together and then back-crossing to C57BL/6J mice to identify recombined Mmu16, carrying both genetic changes on the same chromosome. Mice with the recombined Mmu16 were then crossed with *App^NL-F/+^* animals to generate *Dp3Tyb;App^NL-F/NL-F^* progeny. For this cross *App^NL-F/NL-F^* controls were generated from *App^NL-F/+^* x *App^NL-F/+^* matings, in addition to rare re-recombinations resulting in offspring without the Dp3Tyb segmental duplication but two copies of the *App* knock-in allele. Dp3Tyb controls were generated from Dp3Tyb x C57BL/6J matings generated at the same time as the Dp3Tyb*;App^NL-F/NL-F^* mice. Wild-type (WT) controls were taken from all three matings.

Animals were euthanized by exposure to rising carbon dioxide, followed by confirmation of death by dislocation of the neck in accordance with the Animals (Scientific Procedures) Act 1986 (United Kingdom).

### Tissue preparation and western blotting

For analysis of protein abundance in the hippocampus and cortex, tissues were dissected under ice cold phosphate-buffered saline (PBS) before snap freezing. Samples were then homogenised in RIPA Buffer (150 mM sodium chloride, 50 mM Tris, 1% NP-40, 0.5% sodium deoxycholate, 0.1% sodium dodecyl sulfate) plus complete protease inhibitors (Calbiochem) by mechanical disruption. Total protein content was determined by Bradford assay or Pierce 660 nm assay (Thermo Fisher). Samples from individual animals were analysed separately and were not pooled.

Equal amounts of total brain proteins were then denatured in LDS denaturing buffer (Invitrogen) and β-mercaptoethanol, prior to separation by SDS-PAGE gel electrophoresis using precast 4-12% Bis-Tris gels (Invitrogen). Proteins were transferred to nitrocellulose or PVDF membranes prior to blocking in 5% milk/PBST (0.05% Tween 20) or 5-10% bovine serum albumin (BSA)/PBST. Primary antibodies were diluted in 1% BSA/PBST, HRP-conjugated secondary anti-rabbit, anti-mouse and anti-goat antibodies (Dako) were diluted 1:10,000 in 1% BSA/PBST. Linearity of antibody binding was confirmed by a 2-fold dilution series of cortical protein samples. Band density was analysed using Image J. Relative signal of the antibody of interest compared to the internal loading control was then calculated, and the relative signal was then normalized to the mean relative signal of control samples electrophoresed on the same gel. Means of technical replicates were calculated and used for ANOVA, such that biological replicates were used as the experimental unit.

Primary antibodies against C-terminal APP (Sigma A8717, 1:10,000), β-actin (Sigma A5441, 1:60,000), DYRK1A (7D10, Abnova, 1:500) and BACE2 (Abcam ab5670 1:1,000) were used.

### Biochemical fractionation of mouse brain tissues for the analysis of human amyloid-β

Cortical proteins were fractionated as described in Shankar *et al*. (2009). A half cortex was weighed on a microscale and homogenised in 4 volumes of ice-cold Tris-buffered saline (TBS) (50mM Tris-HCl pH 8.0) containing a cocktail of protease and phosphatase inhibitors (Calbiochem) using a handheld mechanical homogeniser and disposable pestles (Anachem). Samples were then transferred to 1.5 ml microfuge tubes (Beckman Coulter #357448), balanced by adding more TBS and centrifuged at 175,000 × g with an RC-M120EX ultracentrifuge (Sorvall) fitted with rotor S100AT5 at 4 °C for 30 mins. Supernatant (the Tris- fraction) was removed and stored at -80 °C. The remaining pellet was homogenised in 5 volumes of ice-cold 1% Triton X-100 (Sigma-Aldrich) in TBS (50mM Tris-HCl pH 8.0), balanced and centrifuged at 175,000 × g for 30 mins at 4 °C. The resultant supernatant (the Triton-soluble fraction) was removed and stored at -80 °C. The pellet was then re-suspended in 8 volumes (by original cortical weight) of TBS (50mM Tris-HCl pH 8.0), containing 5 M guanidine HCl and left overnight at 4 °C on a rocker to ensure full re-suspension, and subsequently stored at -80 °C. A Bradford assay or Pierce 660 nm protein assay (Thermo Fisher) was performed to determine protein concentration.

### Biochemical preparation of mouse brain tissues for the analysis of mouse amyloid-β

A half cortex was weighed on a microscale and homogenised in 3 volumes of ice-cold TBS (50mM Tris-HCl pH 8.0) containing a cocktail of protease and phosphatase inhibitors (Calbiochem) using a handheld mechanical homogeniser and disposable pestles (Anachem) based on the method in reference (Holtta, Hansson et al. 2013). Homogenates were centrifuged at 21130 × g at 4 °C for 1 hour and the resultant supernatant was stored at -80 °C for onward analysis.

### Quantification of Aβ abundance by Meso Scale Discovery Assay

Amyloid-β_38_, amyloid-β_40_ and amyloid-β_42_ levels were quantified on Multi-Spot 96 well plates pre-coated with anti-amyloid-β_38_, amyloid-β_40_ and amyloid-β_42_ antibodies using multiplex MSD technology, as described in (Wiseman, Pulford et al. 2018). A 6E10 detection antibody was used to quantify human amyloid-β and 4G8 detection antibody for the quantification of mouse amyloid-β. Amounts of amyloid-β_38_, amyloid-β_40_ and amyloid-β_42_ were normalised to the original starting weight of cortical material.

### Immunohistochemistry

Half brains were immersion fixed in 10% buffered formal saline (Pioneer Research Chemicals) for a minimum of 48 hours prior to being processed to wax (Leica ASP300S tissue processor). The blocks were trimmed laterally from the midline by ~0.9-1.4 mm to give a sagittal section of the hippocampal formation. Two 4 μm sections at least 40 μm apart were analysed. The sections were pre-treated with 98% formic acid for 8 mins, followed by washing. The slides were wet loaded onto a Ventana XT for staining (Ventana Medical Systems, Tucson, AZ, USA). The protocol included the following steps: heat induced epitope retrieval (mCC1) for 30 mins in Tris Boric acid EDTA buffer (pH 9.0), superblock (8 mins) and manual application of 100 μl directly biotinylated mouse monoclonal IgG1 antibody against the N-terminus of Aβ (82E1, IBL, 0.2 μg/ml) for 8 hours. Staining was visualised using a ChromoMap DAB kit followed by counterstaining with haematoxylin. The sections were dehydrated, cleared, and mounted in DPX prior to scanning (Leica SCN400F slide scanner). All images were analysed using ImageJ and by manual plaque counting by two independent researchers.

### Biochemical preparation of mouse brain tissues for mass spectrometry of amyloid-β

A half cortex was weighed on a microscale and was homogenized in 5 volumes of tris(hydroxymethyl)aminomethane (Tris)-buffered saline (TBS), pH 7.6, containing cOmplete™ Protease Inhibitor Cocktail (Roche, cat: 04693116001). For the homogenization, one 5 mm bead per sample was used in a TissueLyser (Qiagen) for 4 min at 30 Hz. After homogenization, additional TBS with protease inhibitor cocktail was added up to 1 ml and transferred to a new tube to be centrifuged at 31,000 *g* for 1 h at 4 °C. The pellet was resuspended in 1 ml of 70% formic acid (FA) (v/v), followed by further homogenization in the TissueLyser for 2 min at 30 Hz and subsequent sonication for 30 s. The homogenate was centrifuged again at 31,000 *g* for 1 h at 4 °C and the supernatant (FA fraction) was dried down in a vacuum centrifuge.

Initially, 400 μl of 70% FA (v/v) was added to the dried FA fractions, shaken for 30 min at 21°C and centrifuged at 31,000 *g* for 1 h at4 °C. After the removal of the supernatant, neutralization with 8 ml 0.5 M Tris was performed. Immunoprecipitation (IP) was performed as previously described with some modifications (Gkanatsiou, Portelius et al. 2019). Briefly, 50 μl of sheep anti-mouse magnetic beads (Thermo Fisher Scientific) that had previously been linked with 4 μg each of mouse monoclonal 6E10 and 4G8 antibodies (Biolegend) were added to the neutralized FA fraction. This complex was incubated overnight at 4 °C in 0.2% Triton X-100 in PBS (v/v). By using an automated magnetic-particle KingFisher ml system (Thermo Fisher Scientific), the samples were then washed with PBS Triton X-100, PBS, and 50 mM ammonium bicarbonate separately before elution in 100 μl 0.5% FA. Eluates were dried down in a vacuum centrifuge and stored at -80 °C pending mass spectrometry (MS) analysis.

### Mass spectrometry

Liquid chromatography-mass spectrometry (LC-MS) was conducted in a similar manner as described previously (Portelius, Tran et al. 2007). Briefly, a nanoflow liquid chromatograph was coupled to an electrospray ionization (ESI) hybrid quadrupole–orbitrap tandem MS (Dionex Ultimate 3000 system and Q Exactive, both Thermo Fisher Scientific). Samples were reconstituted in 7 μl 8% FA/8% acetonitrile in water (*v/v/v*) and loaded onto an Acclaim PepMap 100 C18 trap column (length 20 mm; inner diameter 75 μm; particle size 3 μm; pore size 100 Å) for online desalting, and thereafter separated on a reversed-phase Acclaim PepMap RSLC column (length 150 mm, inner diameter 75 μm; particle size 2 μm; pore size 100 Å) (both Thermo Fisher Scientific). Mobile phases were A: 0.1% FA in water *(v/v)*, and B: 0.1% FA/84% acetonitrile in water (*v/v/v*). The flow rate was 300 nl/min and a linear gradient of 3-40% B for 50 min was applied. The temperature of the column oven was 60 °C. Mass spectrometer settings were as follows: positive ion mode; mass-to-charge *(m/z)* interval 350-1800 *m/z* units; data dependent acquisition with 1 precursor ion acquisition (MS) followed by up to 5 fragment ion acquisitions (MS/MS); resolution setting 70,000 (for both MS and MS/MS); number of microscans 1 (MS and MS/MS); target values 10^6^ (MS and MS/MS); maximum injection time 250 ms (MS and MS/MS); fragmentation type was higher-energy collisional dissociation fragmentation (HCD); normalised collision energy (NCE) setting 25; singly charged ions and ions with unassigned charge were excluded for MS/MS selection. Database search (including isotope and charge deconvolution) and label free quantification was performed with PEAKS Studio v8.5 (Bioinformatics Solutions Inc.) against a custom-made APP database. All suggested fragment mass spectra were evaluated manually.MS signal was normalised to starting weight of the cortex prior to data analysis.

### Statistical analysis and experimental design

All experiments and analyses were undertaken blind to genotype and sex. A 6-digit identification number was allocated to each animal prior to genotyping which was used to blind samples. Experimental groups for all experiments were pseudorandomised using Mendelian inheritance. Mice not carrying the correct combination of alleles (i.e. *App^NL-F/+^* heterozygous mice) were excluded from the analysis, no other animals were excluded. Some samples were lost from the study because of technical issues during tissue processing to wax or during fractionation. Data were analysed as indicated in figure legends by univariate ANOVA with between-subject factors being genetic status (*App^+/+^/App^NL-F/NL-F^* and/or wild-type/Hsa21 or wild-type/Dpx) and sex. Fractionation batch was included as a between-subject factor for analysis of amyloid-β by MSD assays. The subject means of technical replicates were calculated and used in the ANOVA for western blot and MSD assays, as the number of replicates for which data was available varied between samples. Repeat measures ANOVA was used for manual plaque counts (combining the data of two independent researchers).

## Results

### Trisomy of chromosome 21 decreases accumulation of amyloid-β in the cortex of the *App^NL-F^* mouse model

**Figure 2:**
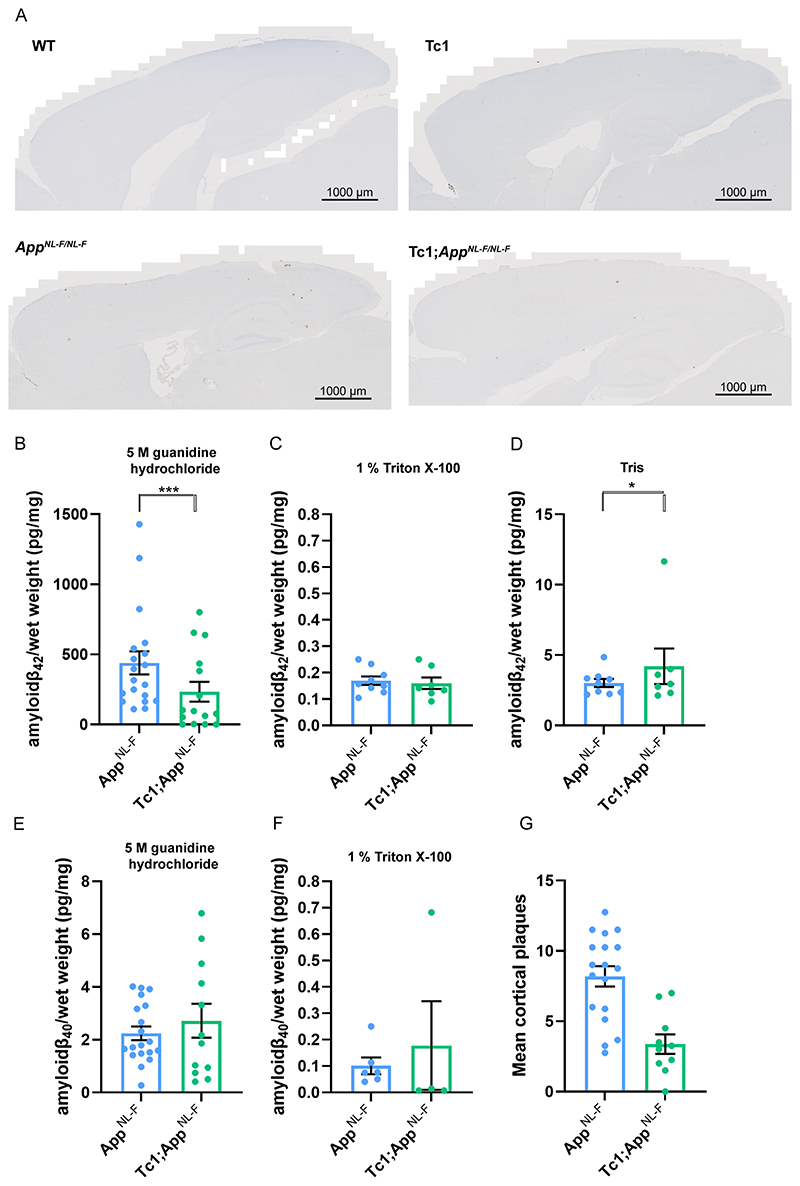
Trisomy of Hsa21 results in a decrease in amyloid-β deposition in the *App^NL-F/NL-F^* model. **(A, G)** Amyloid-β deposition (82E1) in the cortex was quantified at 8-months of age in male and female mice by manual plaque counting. **(A)** Representative image of 82E1 stained amyloid-β deposits (brown) in wild-type (WT), Tc1, *App^NL-F/NL-F^*, Tc1;*App^NL-F/NL-F^* mice. **(G)** Significantly fewer amyloid-β deposits in the cortex were observed in Tc1;*App^NL-F/NL-F^* compared with *App^NL-F/NL-F^* controls (F(1,23) = 24.997, p < 0.001). *App^NL-F/NL-F^* female n = 11, male n = 7, Tc1;*App^NL-F/NL-F^* female n = 4, male n = 6. No amyloid-β deposits were observed in WT (female n = 5) or Tc1 (female n = 5, male n = 3) age matched littermate controls (data not shown). (**B-F)** Total cortical proteins were biochemically fractionated and amyloid abundance analysed by MSD assay. **(B)** No statistically significant difference in the abundance of 5 M guanidine hydrochloride soluble amyloid-β_42_ was observed between Tc1;*App^NL-F/NL-F^* compared with *App^NL-F/NL-F^* controls (F(1,13) = 2.262, p = 0.156). *App^NL-F/NL-F^* female n = 15, male n = 8; Tc1*;App^NL-F/NL-F^* female n = 8, male n = 10. WT and Tc1 samples which do not produce human amyloid-β were included as negative controls. WT n = 4, of which 1 was below limit of detection ($\bar{\text{x}}=0.543$ pg/mg, SEM = 0.433); Tc1 n = 6, of which 4 samples were below the limit of detection ($\bar{\text{x}}=2.175$ pg/mg, SEM 1.437). **(C)** No difference in the abundance of 1% Triton X-100 soluble amyloid-β_42_ was observed between Tc1;*App^NL-F/NL-F^* compared with *App^NL-F/NL-F^* controls (F(1,5) = 0.015, p = 0.907). *App^NL-F/NL-F^* female n = 7, male n = 2 (n = 8 below limit of detection); Tc1*;App^NL-F/NL-F^* female n = 5, male n = 2 (n = 11 below limit of detection). WT and Tc1 samples which do not produce human amyloid-β were included as negative controls. WT n = 4, of which 2 were below the limit of detection ($\bar{\text{x}}=0.021$ pg/mg, SEM = 0.007); Tc1 n = 6, of which all samples were below the limit of detection. **(D)** Significantly more Tris soluble amyloid-β_42_ was observed in Tc1; *App^NL-F/NL-F^* compared with *App^NL-F/NL-F^* controls (F(1,5) = 10.697, p = 0.022). *App^NL-F/NL-F^* female n = 7, male n = 2 (n = 8 below limit of detection) Tc1*;App^NL-F/NL-F^* female n = 5, male n = 2 (n = 11 below limit of detection). WT and Tc1 samples which do not produce human amyloid-β were included as negative controls. WT n = 4, of which all were below the limit of detection; Tc1 n = 6, of which all samples were below the limit of detection. **(E)** No difference in the abundance of 5 M guanidine hydrochloride-soluble amyloid-β_40_ was observed between *Tc1;App^NL-F/NL-F^* compared with *App^NL-F/NL-F^* controls (F(1,13) = 0.005, p = 0.946). *App^NL-F/NL-F^* female n = 11, male n = 8, n = 4 below the limit of detection; Tc1*;App^NL-F/NL-F^* female n = 7, male n = 5, n = 6 below the limit of detection. WT and Tc1 samples which do not produce human amyloid-β were included as negative controls. WT n = 4, of which 3 were below the limit of detection (1.870 pg/mg); Tc1 n = 6, of which 5 samples were below the limit of detection (1.150 pg/mg). **(F)** No difference in the abundance of 1% Triton X-100-soluble amyloid-β_40_ was observed between *Tc1;App^NL-F/NL-F^* compared with *App^NL-F/NL-F^* control (F(1,3) = 0.000, p = 0.991). *App^NL-F/NL-F^* female n = 4, male n = 0 (n = 17 below the limit of detection); Tc1*;App^NL-F/NL-F^* female n = 4, male n = 2 (n = 14 below the limit of detection). Tris-soluble amyloid-β_40_ was not detected in these samples. WT and Tc1 samples which do not produce human amyloid-β were included as negative controls. WT n = 4, of which 2 samples were below limit of detection ($\bar{\text{x}}=0.090$ pg/mg, SEM = 0.005); Tc1 n = 6, of which all samples were below the limit of detection. Error bars show SEM, data points are independent mice.

Following on from our previous studies, which indicated that an additional copy of Hsa21 alters APP biology and the accumulation of amyloid-β *in vivo* in an *APP* transgenic model (Wiseman, Pulford et al. 2018, Tosh, Rhymes et al. 2021), here we determined if an additional copy of Hsa21 modulated APP/amyloid-β biology in the *App^NL-F^* knock-in mouse model. We undertook a two generation cross of the Tc1 mouse model of DS (O’Doherty, Ruf et al. 2005), which contains a freely segregating copy of Hsa21 (but not a functional additional copy of *APP*) (Gribble, Wiseman et al. 2013), with the *App^NL-F^* model to generate 4 genotypes of mice (wild-type, Tc1, *App^NL-F/NL-F^* and *Tc1;App^NL-F/NL-F^)*. We quantified the number of 82E1^+^ amyloid-β deposits in the cortex of these 4 genotypes of mice at 8-months of age. 82E1^+^ deposits were not observed in wild-type or Tc1 mice consistent with our previous study (Wiseman, Pulford et al. 2018). We found a significant decrease in the number of deposits in Tc1;*App^NL-F/NL-F^* compared with *App^NL-F/NL-F^* controls (Fig. 2 A, G).

We also determined if trisomy of Hsa21 modulated the biochemical aggregation of amyloid-β_40_ and amyloid-β_42_ in the cortex at 8-months of age, using biochemical protein fractionation by step-wise homogenisation and ultracentrifugation in sequentially more disruptive solutions (Tris-HCl, Tris-HCl 1% Triton X-100 and finally 5 M guanidine hydrochloride). We then quantified human amyloid-β_40_ and amyloid-β_42_ in each fraction normalised to starting brain weight (6E10 MSD triplex assay). Samples from mice without a humanised *App* allele, that do not produce human amyloid-β, were used as negative controls. Amyloid-β_42_ in the guanidine hydrochloride fraction was not significantly reduced in the presence of the extra copy of Hsa21 (Fig. 2B). A significant increase in Tris-soluble amyloid-β_42_ was seen in the cortex of *Tc1;App^NL-F/NL-F^* compared with *App^NL-F/NL-F^* controls (Fig. 2D). However, we note that this effect is being driven by one outlier and significance is lost if this animal is excluded from the analysis moreover this analyte could not be detected in a significant proportion of the samples analysed. Thus a replication study is required to determine the validity of this result. No significant difference in Triton-soluble amyloid-β_42_ abundance was observed (Fig. 2C). The amount of human amyloid-β_40_ in the *App^NL-F/NL-F^* model is very low because of the Iberian mutation in the modified *App* allele and this analyte was below the limit of detection in the Tris-soluble fraction and did not significantly differ between genotypes in the Triton and 5 M guanidine hydrochloride fractions (Fig. 2E, F). These data indicate that an additional copy of a Hsa21 gene or genes is sufficient to reduce the deposition of amyloid-β in the cortex, this may occur via an effect on amyloid-β formation, clearance or aggregation,

### Decreased amyloid-β accumulation in the *Tc1-App^NL-F/NL-F^* model does not occur because of a reduction of APP or CTF-β abundance

**Figure 3:**
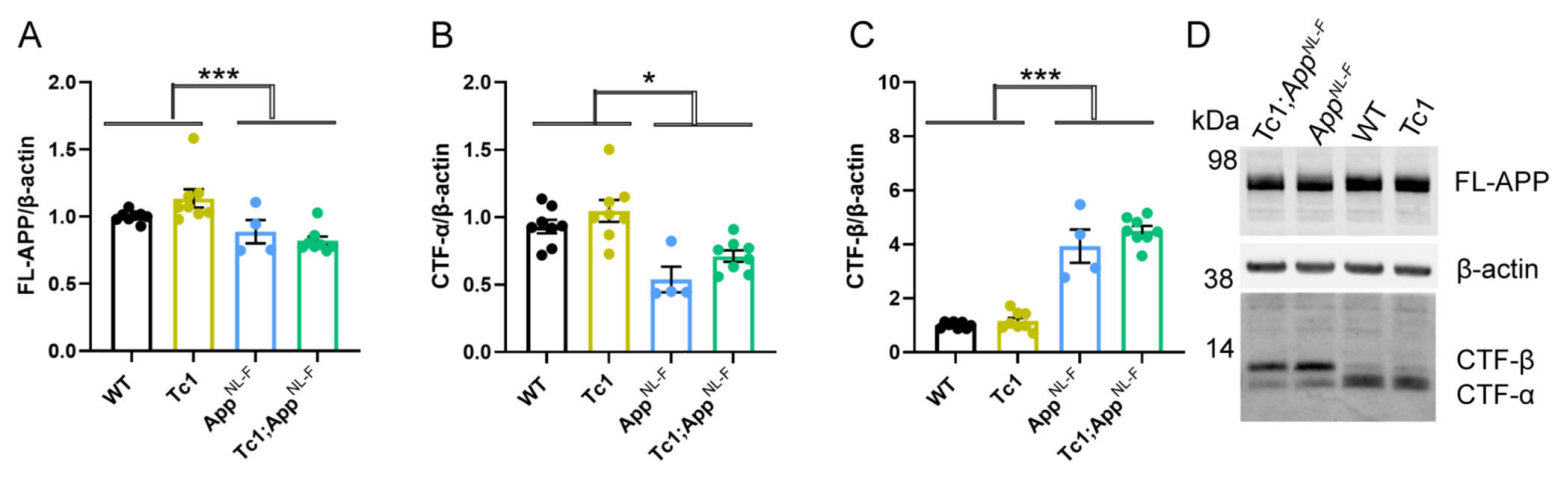
The abundance of FL-APP and CTF is not altered by trisomy of Hsa21. **(A-D)** The relative abundance of full-length APP (FL-APP), APP β-C-terminal fragment (β-CTF) and APP α-C-terminal fragment (α-CTF) compared to β-actin was measured by western blot using A8717 primary antibody in the cortex at 3-months of age in female and male mice. **(A)** Significantly less FL-APP was observed in mice in which *App* was humanised and mutated (F(1,19) = 23.837, p < 0.001). An additional copy of Hsa21 did not alter FL-APP abundance (F(1,19) = 0.599, p = 0.449). **(B)** Significantly less CTF-α was observed in mice in which *App* was humanised and mutated (F(1,19) = 5.950, p = 0.025) but an additional copy of Hsa21 did not alter α-CTF abundance (F(1,19) = 3.012, p = 0.099). **(C)** Significantly more β-CTF was observed in mice in which *App* was humanised and mutated (F(1,19) = 868.431, p < 0.001). By ANOVA a significant effect of Hsa21 on CTF-β abundance was detected (F(1,19) = 23.462, p < 0.001), however wild-type (WT) and Tc1 (Bonferroni pair-wise comparison p = 1.000) and *App^NL-F/NL-F^* and Tc1*;App^NL-F/NL-F^* (Bonferroni pair-wise comparison p = 0.118) were not statistically significant. WT female n = 4, male n = 4: Tc1 female n = 3, male n = 5; *App^NL-F/NL-F^* female n = 2, male n = 2; Tc1*;App^NL-F/NL-F^* female n = 4, male n = 4. **(D)** Representative image of western blot in WT, Tc1, *App^NL-F/NL-F^*, Tc1;*App^NL-F/NL-F^* mice. Error bars show SEM, data points are independent mice.

The significant increase in Tris-soluble amyloid-β_42_ in the Tc1;*App^NL-F/NL-F^* cortex suggests that the decrease in amyloid-β accumulation observed is likely to be caused by a change in peptide aggregation. However, previous studies have suggested that genes on Hsa21, other than *APP*, can increase APP protein level *in vivo* and modulate the abundance of the amyloid-β precursor APP-C-terminal fragment-β (CTF-β) (Garcia-Cerro, Rueda et al. 2017, Naert, Ferre et al. 2018, Wiseman, Pulford et al. 2018). Thus, we used western blotting to determine if the additional Hsa21 genes altered APP or CTF-β abundance in our new model system. Here, we found no evidence of decreased mouse or human full-length APP (FL-APP) in the cortex of the Tc1 and *Tc1;App^NL-F/NL-F^* compared with wild-type and *App^NL-F/NL-F^* mice (Fig. 3A, D). We note that significantly less FL-APP is detected in humanised models compared with controls (using antibody A8717), this may reflect a reduction in antibody binding rather than a biological reduction in protein level.

We found a significant increase in CTF-β and a significant decrease in CTF-α in the cortex of *App^NL-F/NL-F^* mice consistent with the reported effects of the introduced mutations on APP processing (Fig. 3B, C, D) (Saito, Matsuba et al. 2014). An additional copy of Hsa21 did not significantly alter wild-type APP CTF-β or CTF-α levels in the cortex of the Tc1 compared to wild-type-mice or in the *Tc1;App^NL-F/NL-F^* compared to *App^NL-F/NL-F^*. This finding contrasted with the large increase in CTF-β in male Tc1;*APP* transgenic model that we previously reported (Wiseman, Pulford et al. 2018). These data suggest that the reduction in deposition of amyloid-β in the Tc1;*App^NL-F/NL-F^* model is likely mediated by an enhancement of amyloid-β clearance or an impairment of peptide aggregation rather than a decrease in APP or CTF-β abundance.

### Decreased accumulation of amyloid-β is caused by an additional copy of 38 Hsa21 orthologous genes

Many DS-associated phenotypes are multigenic, caused by the combined effect of multiple Hsa21 genes acting together on one biological pathway. To understand the mechanisms underlying the decrease in amyloid-β accumulation in the Tc1*;App^NL-F/NL-F^* model further, we used a series of mouse models of DS that carry an extra copy of subregions of mouse chromosomes that are orthologous with Hsa21, to identify the combination of regions/genes responsible for the changes (Fig. 1) (Yu, Li et al. 2010, Lana-Elola, Watson-Scales et al. 2016). We used 3 mouse lines that carry similar gene content to the Tc1 mouse model. However, because of the limitations of available models we were unable to explore the Hsa21 genes closest to *App* on Mmu16, which are in three copies in the Tc1 model, as using recombination to generate the required combination of alleles in the available Dp9Tyb model was not feasible.

**Figure 4:**
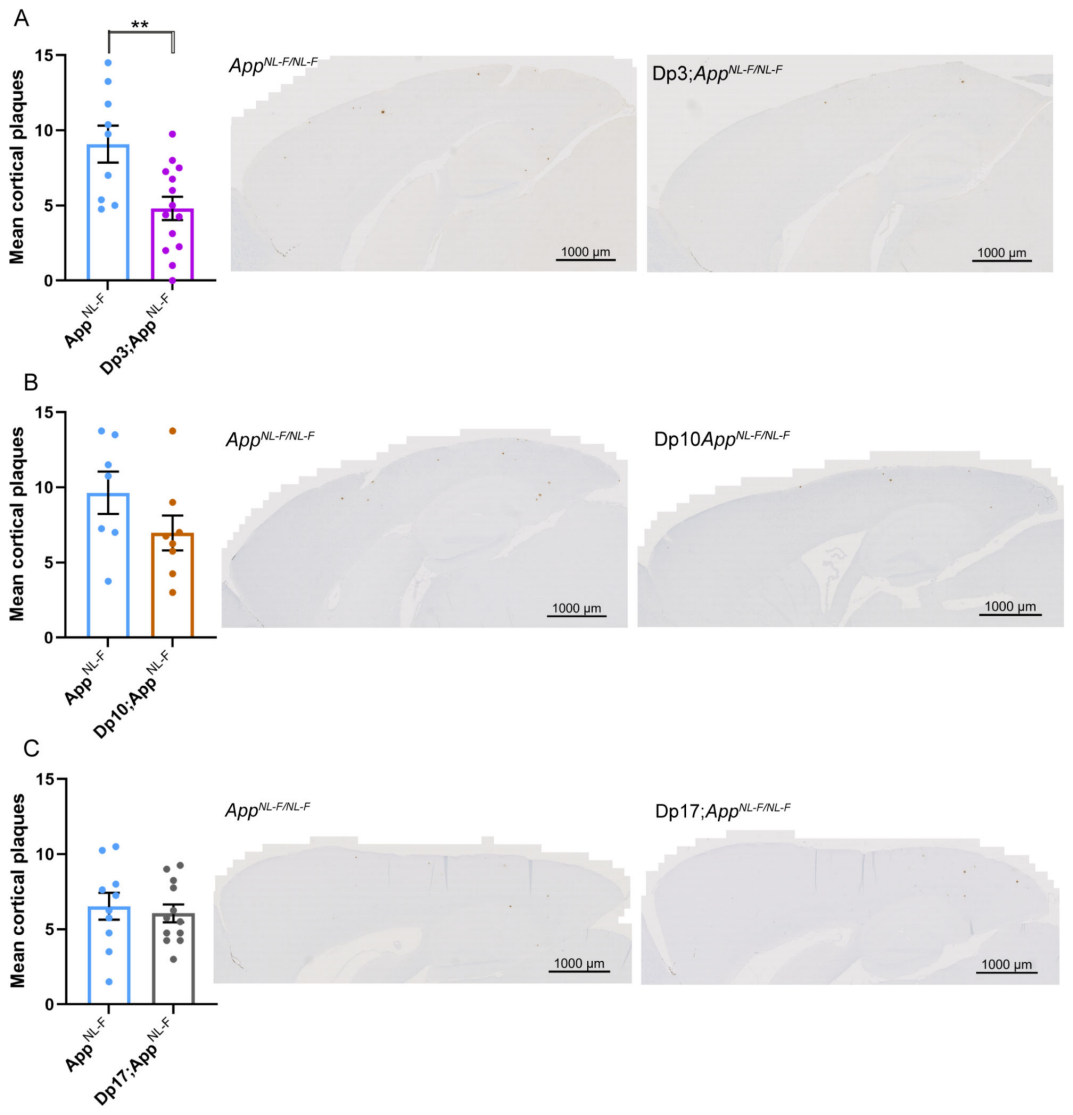
Duplication of the Dp3Tyb region of mouse chromosome 21 orthologous with Hsa21 is sufficient to decrease amyloid-β accumulation in the *App^NL-F/NL-F^* model. **(A-C)** Amyloid-β deposition (82E1) in the cortex was quantified at 8-months of age in male and female mice by manual plaque counting. **(A)** Significantly fewer amyloid-β deposits were observed in the cortex of Dp3Tyb;*App^NL-F/NL-F^* compared with *App^NL-F/NL-F^* controls (F(1,18) = 12.359, p = 0.002). *App^NL-F/NL-F^* female n = 2, male n = 7; Dp3Tyb;*App^NL-F/NL-F^* female n = 9, male n = 5. No amyloid deposits were observed in n = 4 WT controls. **(B)** A non-significant trend for reduced amyloid-β deposits in the cortex was observed in Dp(10)2Yey;*App^NL-F/NL-F^* compared with *App^NL-F/NL-F^* controls (F(1,11) = 12.359, p = 0.077). *App^NL-F/NL-F^* female n = 4, male n = 3; Dp(10)2Yey;*App^NL-F/NL-F^* female n = 6, male n = 2. No amyloid-β deposits were observed in WT n = 6 and Dp(10)2Yey n = 3 controls. **(C)** No difference in amyloid-β deposits were observed in the cortex of Dp(17)3Yey;*App^NL-F/NL-F^* compared with *App^NL-F/NL-F^* controls (F(1,18) = 0.021, p = 0.885). *App^NL-F/NL-F^* female n = 6, male n = 4; Dp(17)3Yey *App^NL-F/NL-F^* female n = 6, male n = 6. No amyloid-β deposits were observed in WT n = 6 and Dp(17)3Yey n = 6 controls. Dp3Tyb abbreviated to Dp3, Dp(10)2Yey abbreviated to Dp10, Dp(17)3Yey abbreviated to Dp17 for clarity. Error bars show SEM, data points are independent mice

An extra copy of the genes between *Mir802* and *Zbtb21* (Dp3Tyb) was sufficient to decrease the accumulation of amyloid-β in the cortex, as quantified by 82E1 plaque counts (Fig. 4A). However, an extra copy of the genes between *Abcg1* and *Rrp1b* (Dp(10)2Yey) and between *Prmt2* and *Pdxk* (Dp(17)3Yey) was not sufficient to significantly alter the accumulation of amyloid-β in the cortex, as quantified by 82E1 plaque counts in 8-month old mice (Fig. 4B, C).

The abundance of amyloid-β_42_ in the guanidine hydrochloride fraction was not significantly altered in the Dp3Tyb;*App^NL-F/NL-F^*, Dp(10)2Yey;*App^NL-F/NL-F^* or

**Figure 5:**
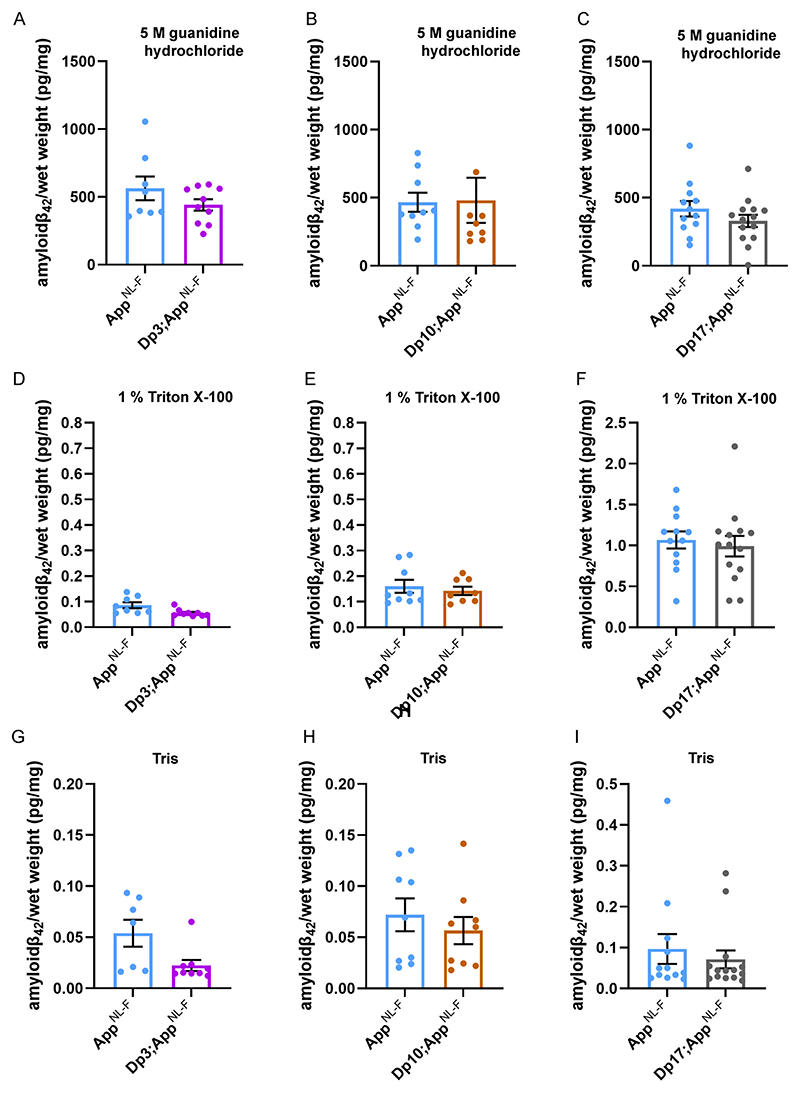
Biochemical solubility of amyloid-β_42_ is not altered by an additional copy of the Dp3Tyb, Dp(10)2Yey or Dp(17)3Yey Hsa21 orthologous regions. Total cortical proteins were biochemically fractionated from 8-month of age mice and amyloid-β abundance analysed by MSD assay (6E10). No difference in the abundance of **(A)** 5 M guanidine hydrochloride soluble amyloid-β_42_ (F(1,12) = 0.128, p = 0.726), **(D)** 1% Triton X-100 soluble amyloid-β_42_ (F(1,12) = 2.863, p = 0.116) or **(G)** Tris-soluble amyloid-β_42_ (F(1,10) = 0.281, p = 0.608) was observed between *Dp3Tyb; App^NL-F/NL-F^* compared with *App^NL-F/NL-F^* controls. *App^NL-F/NL-F^* female n = 2, male n = 6; *Dp3Tyb;App^NL-F/NL-F^* female n = 7, male n = 3. No difference in the abundance of **(B)** 5 M guanidine hydrochloride soluble (F(1,11) = 2.337, p = 0.115), **(E)** 1% Triton X-100-soluble amyloid-β_42_ (F(1,11) = 0.145, p = 0.711) or **(H)** Tris-soluble amyloid-β_42_ (F(1,11) = 0.001, p = 0.978) was observed between Dp(10)2Yey;*App^NL-F/NL-F^* compared with *App^NL-F/NL-F^* controls. *App^NL-F/NL-F^* female n = 4, male n = 5; Dp(10)2Yey*;App^NL-F/NL-F^* female n = 6, male n = 3. No difference in the abundance of **(C)** 5 M guanidine hydrochloride soluble (F(1,20) = 4.079, p = 0.057), **(F)** 1% Triton X-100 soluble amyloid-β4_2_ (F(1,20) = 0.124, p = 0.728) or **(I)** Tris soluble (F(1,20) = 0.521, p = 0.479) amyloid-β_42_ was observed between Dp(17)3Yey;*App^NL-F/NL-F^* compared with *App^NL-F/NL-F^* controls. *App^NL-F/NL-F^* female n = 7, male n = 5; Dp(17)3Yey*;App^NL-F/NL-F^* female n = 7, male n = 7. Details of negative controls, which do not carry an *App^NL-F^* allele in Table 2. Dp3Tyb abbreviated to Dp3, Dp(10)2Yey abbreviated to Dp10, Dp(17)3Yey abbreviated to Dp17 for clarity. Error bars show SEM, data points are independent mice.

**Figure 6:**
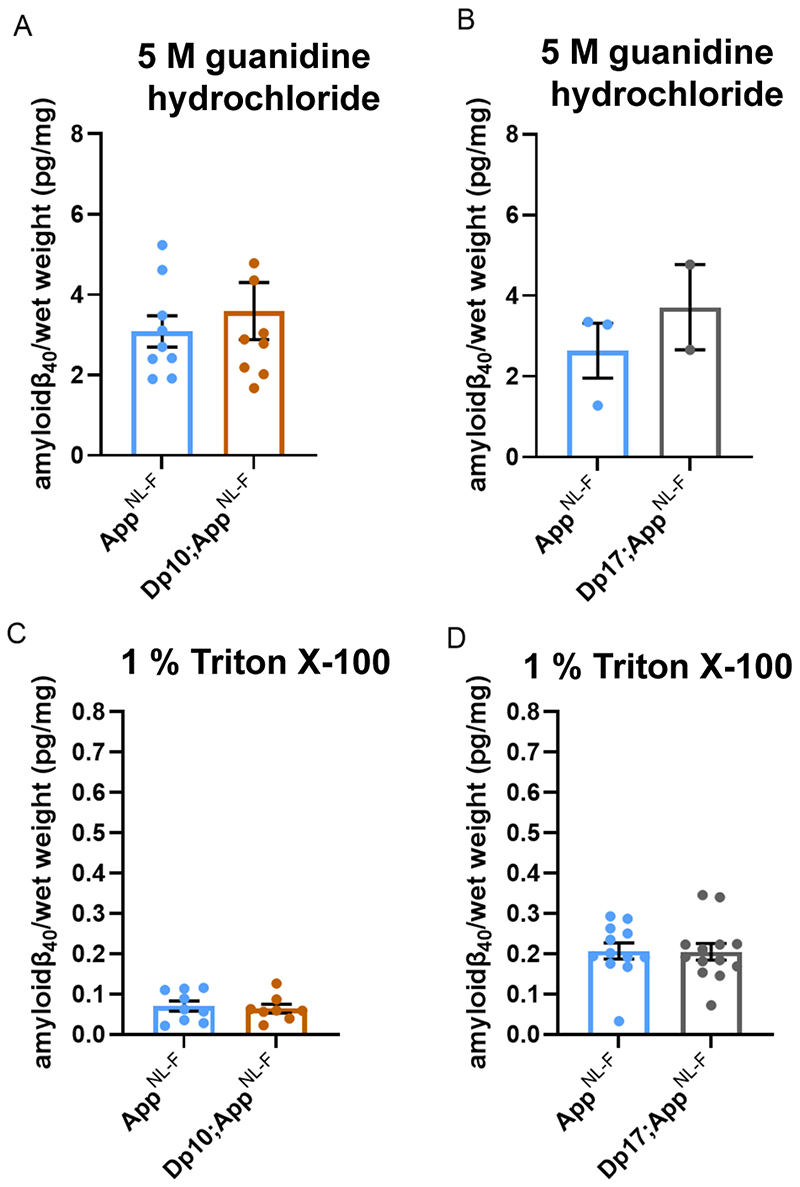
Biochemical solubility of amyloid-β_40_ is not altered by an additional copy of the Dp(10)2Yey or Dp(17)3Yey Hsa21 orthologous regions. Total cortical proteins were biochemically fractionated from 8-month of age mice and amyloid-β abundance analysed by MSD assay (6E10). **(A)** No difference in the abundance of 5 M guanidine hydrochloride-soluble amyloid-β_40_ was observed between Dp(10)2Yey;*App^NL-F/NL-F^* compared with *App^NL-F/NL-F^* controls (F(1,12) = 2.137, p = 0.169). *App^NL-F/NL-F^* female n = 6, male n = 3; Dp(10)2Yey*;App^NL-F/NL-F^* female n = 4, male n = 5. **(B)** No difference in the abundance of 5 M guanidine hydrochloride-soluble amyloid-β_40_ was observed between Dp(17)3Yey;*App^NL-F/NL-F^* compared with *App^NL-F/NL-F^* controls (F(1,1) = 0.782, p = 0.539). *App^NL-F/NL-F^* female n = 1, male n = 2, n = 9 below limit of detection; Dp(17)3Yey*;App^NL-F/NL-F^* female n = 2, male n = 0, n = 12 below limit of detection. **(C)** No difference in the abundance of 1% Triton X-100-soluble amyloid-β_40_ was observed between Dp(10)2Yey;*App^NL-F/NL-F^* compared with *App^NL-F/NL-F^* controls (F(1,11) = 0.540, p = 0.478). *App^NL-F/NL-F^* female n = 6, male n = 3; Dp(10)2Yey*;App^NL-F/NL-F^* female n = 4, male n = 4. **(D)** No difference in the abundance of 1% Triton X-100-soluble amyloid-β_40_ was observed between Dp(17)3Yey;*App^NL-F/NL-F^* compared with *App^NL-F/NL-F^* controls (F(1,19) = 0.007, p = 0.982). *App^NL-F/NL-F^* female n = 7, male n = 5; Dp(17)3Yey*;App^NL-F/NL-F^* female n = 7, male n = 6, n = 1 below limit of detection. Details of negative controls, which do not carry an *App^NL-F^* allele in Table 2. Error bars show SEM, data points are independent mice. Dp(10)2Yey abbreviated to Dp10 and Dp(17)3Yey abbreviated to Dp17 for clarity.

Dp(17)3Yey;*App^NL-F/NL-F^* mice compared with *App^NL-F/NL-F^* controls at 8-months of age (Fig. 5A-C). No significant difference in the abundance of Tris- or Triton-soluble amyloid-β_42_ was observed in Dp3Tyb;*App^NL-F/NL-F^*, Dp(10)2Yey;*App^NL-F/NL-F^* or Dp(17)3Yey;*App^NL-F/NL-F^* compared to controls (Fig. 5D-I). No difference in the abundance of guanidine hydrochloride or Triton-soluble amyloid-β_40_ was observed between Dp(10)2Yey;*App^NL-F/NL-F^* or Dp(17)3Yey;*App^NL-F/NL-F^* compared to controls (Fig. 6A-D). These data indicate that a gene or genes in 3-copies in the Dp3Tyb model is sufficient to decrease the deposition of amyloid-β in the brain.

### Increased DYRK1A does not lead to increased APP or amyloid-β in the Dp3Tyb model of DS

**Figure 7:**
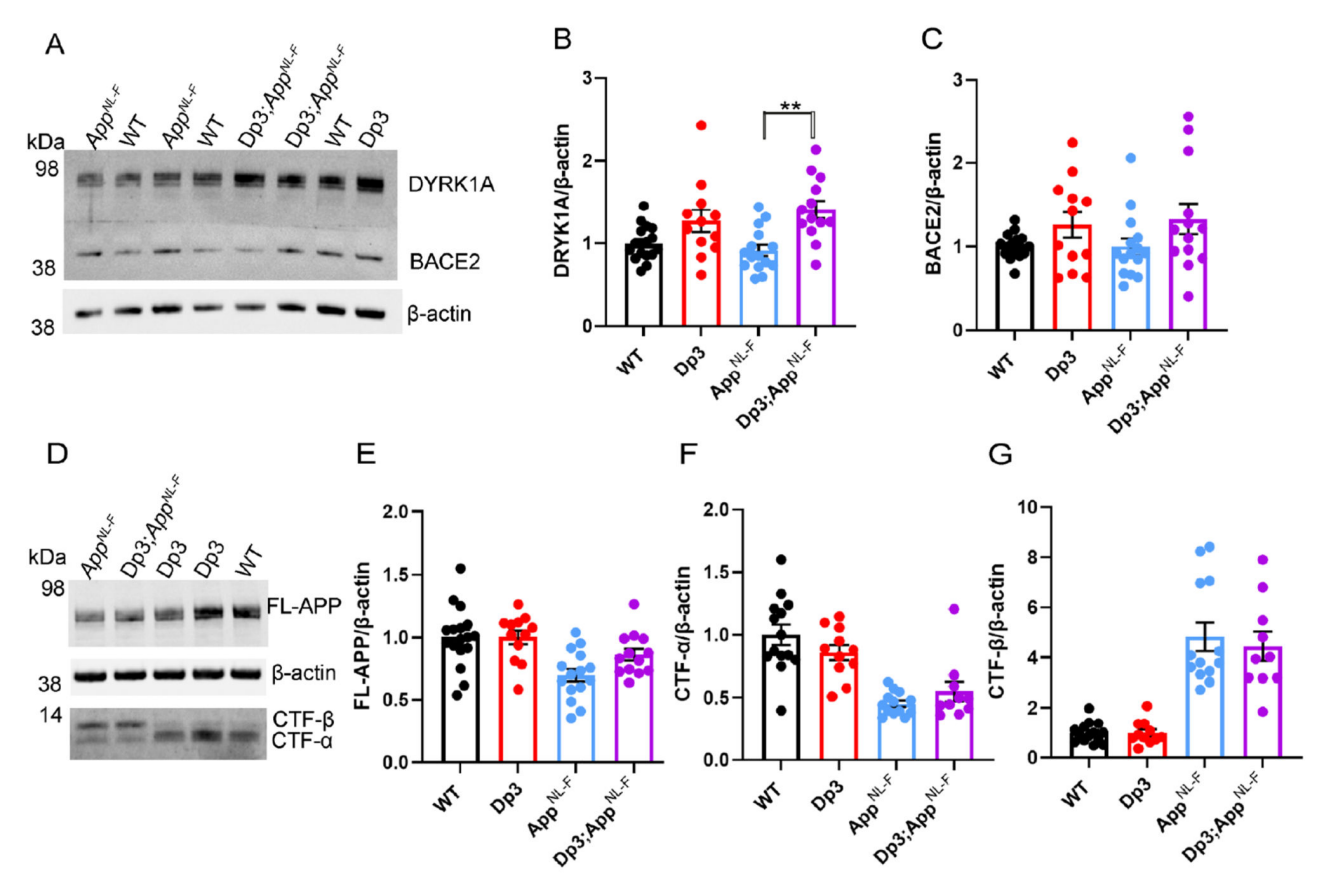
Duplication of the Dp3Tyb region is sufficient to raise the protein abundance of DYRK1A in the cortex but does not significantly alter BACE2, FL-APP or CTF abundance. The abundance of **(A-B)** DYRK1A, **(A, C)** BACE2, **(D, E)** full-length, APP (FL-APP), **(D, F)** C-terminal fragment-α (CTF-α) and **(D, G)** C-terminal fragment-β (CTF-β) relative to β-actin loading control was measured by western blot in the cortex at 3 months of age in male and female mice. ANOVA analysis indicated that an additional copy of the Dp3Tyb region increased the abundance of **(B)** DYRK1A (F(1,49) = 16.511, p < 0.001) and **(C)** BACE2 F(1,49) = 4.444, p = 0.040). Post-hoc pair-wise comparison with Bonferroni correction for multiple comparison, demonstrated that significantly higher levels of DYRK1A were oberved in *Dp3Tyb;App^NL-F/NL-F^* compared to *App^NL-F/NL-F^* cortex (p = 0.002) but that BACE2 levels did not differ between these two genotypes of mice (p = 0.359). There was no effect of an extra copy of the Dp3Tyb region on the abundance of **(E)** FL-APP level (F(1,49) = 2.183, p = 0.126), **(F)** CTF-α (F(1,40) = 0.040, p = 0.843), or **(G)** CTF-β (F(1,40) = 0.008, p = 0.929). As previously reported (Saito, Matsuba et al. 2014), mice homozygous for the *App^NL-F^* allele had lower abundance of **(E)** FL-APP (F(1,49) = 16.790, p < 0.001), **(F)** CTF-α (F(1,40) = 15.739, p < 0.001) and a higher abundance of **(G)** CTF-β (F(1,40) = 147.440, p < 0.001). Wild-type (WT) (female n = 6, male n = 11), Dp3Tyb (female n = 5, male n = 7), *App^NL-F^* (female n = 8, male n = 7) and Dp3Tyb*;App^NL-F/NL-F^* (female n = 5, male n = 8). CTF were below the limit of detection in Wild-type (n = 3) Dp3Tyb (n = 1), *App^NL-F^* (n = 2) and *Dp3Tyb;App^NL-F/NL-F^* (n = 3) samples. Error bars show SEM, data points are independent mice. Dp3Tyb abbreviated to Dp3 for clarity.

The Dp3Tyb model contains an additional copy of 38 genes, including *Dyrk1a*, a kinase that phosphorylates APP (Ryoo, Cho et al. 2008), increasing the abundance of the protein *in vivo*, contributing to raised soluble amyloid-β abundance in the Ts65Dn mouse model of DS (Garcia-Cerro, Rueda et al. 2017). We wanted to determine whether we could observe this previously reported biology in our new model system. We found that an extra copy of the Dp3Tyb region raises the abundance of DYRK1A in the cortex of 3-month-old animals, including in the context of *App^NL-F^* knock-in mutations (Fig. 7A-B). This is consistent with numerous previous reports of dosage sensitivity of DYRK1A in the mouse throughout lifespan (Sheppard, Plattner et al. 2012, Garcia-Cerro, Rueda et al. 2017, Yin, Jin et al. 2017). We also note that consistent with previous reports in other mouse model systems, 3-copies of *Dyrk1a* in the *Dp3Tyb;App^NL-F/NL-F^* model was associated with an increase in cortical weight (Guedj, Pereira et al. 2012) (Fig. 10A).

**Figure 8:**
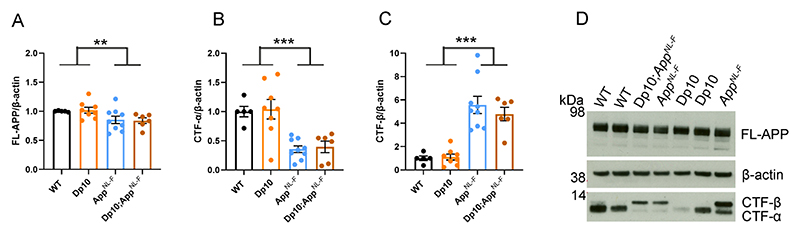
The abundance of FL-APP and CTF is not altered by duplication of the Dp10Yey Hsa21 orthologous region The abundance of **(A, D)** full-length, APP (FL-APP), **(B, D)** C-terminal fragment-α (CTF-α) and **(C, D)** C-terminal fragment-β (CTF-β) relative to β-actin loading control was measured by western blot in the cortex at 3-months of age in male and female mice. There was no effect of an extra copy of the Dp(10)2Yey region on the abundance of **(A)** FL-APP level (F(1,22) = 0.828, p = 0.372), **(B)** CTF-α (F(1,22) = 0.054, p = 0.819) or **(C)** CTF-β abundance (F(1,22) = 0.829, p = 0.372). As previously observed, mice homozygous for the *App^NL-F^* allele had lower abundance of **(A)** FL-APP (F(1,22) = 8.168, p = 0.009), **(B)** CTF-α (F(1,22) = 72.150, p < 0.001) and a higher abundance of **(C)** CTF-β (F(1,22) = 15.300, p < 0.001). Wild-type (WT) (female n = 1, male n = 4), Dp(10)2Yey (male n = 8), *App^NL-F^* (male n = 9) and *Dp(10)2Yey;App^NL-F/NL-F^* (female n = 2, male n = 4). Error bars show SEM, data points are independent mice. Dp(10)2Yey abbreviated to Dp10 for clarity.

**Figure 9:**
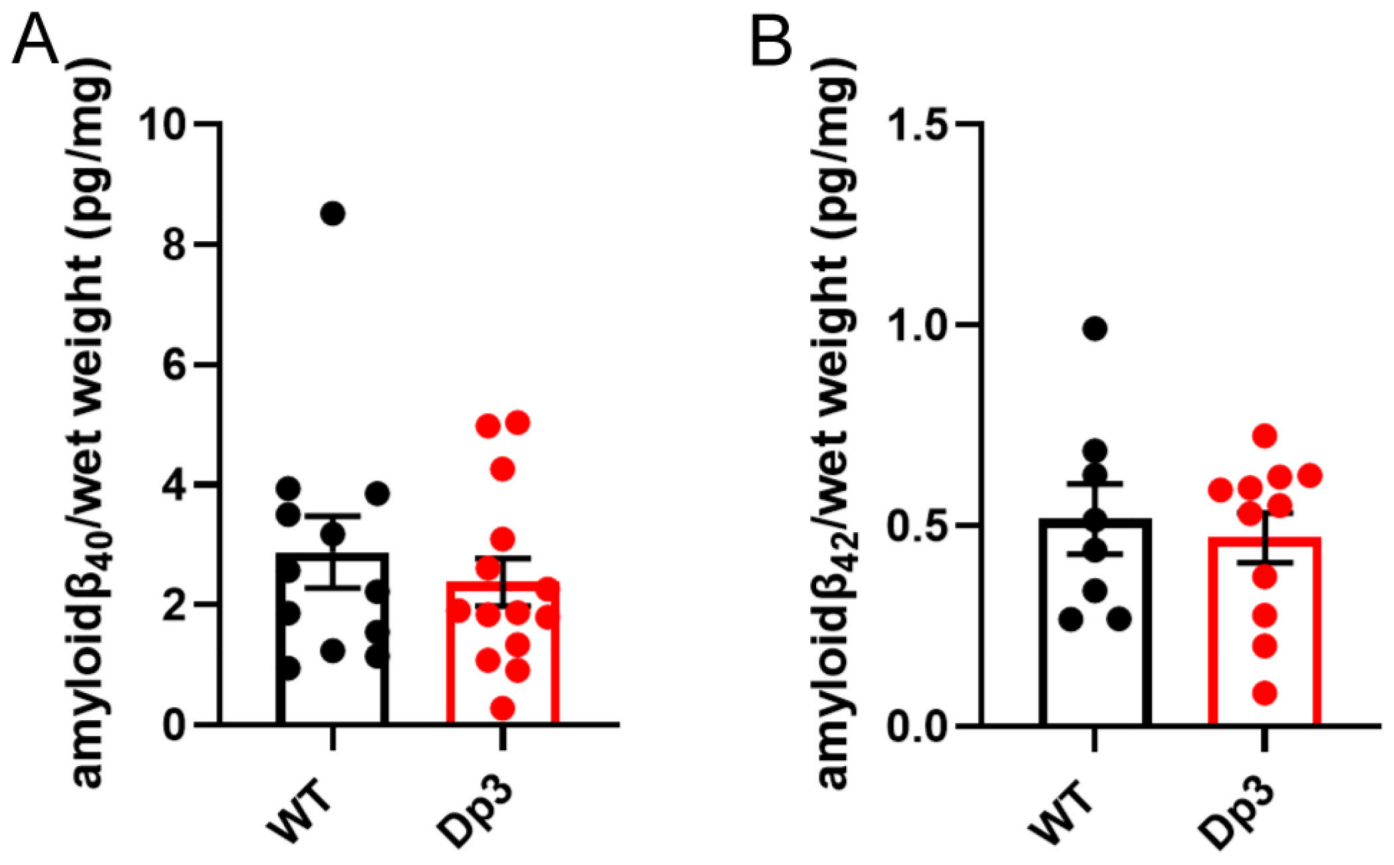
An additional copy of the Dp3Tyb region does not alter the abundance of mouse amyloid-β in the young cortex. Total cortical proteins were homogenised in TBS buffer, from mice of 3-months of age, and amyloid-β abundance was analysed by MSD assay (4G8). An additional copy of the Dp3Tyb region did not alter the abundance of **(A)** amyloid-β_40_ (F1,14) = 0.693, p = 0.419) or **(B)** amyloid-β_42_ (F1,7) = 0.410, p = 0.718). Wild-type (WT) (female n = 5, male n = 11), Dp3Tyb (female n = 7, male n = 12). For amyloid-β_40_ (WT n = 4 and Dp3Tyb = 5) and amyloid-β_42_ (WT= 8 and Dp3Tyb n = 8) were below the limit of detection. Error bars show SEM, data points are independent mice. Dp3Tyb abbreviated to Dp3 for clarity.

However, we found no evidence of changes to the abundance of humanised-APP or mouse-APP in *Dp3Tyb;App^NL-F/NL-F^* or Dp3Tyb models compared to *App^NL-F/NL-F^* controls in the cortex at 3-months of age (Fig. 7D-E). Similarly no change in human-CTF-β or mouse-CTFβ was observed in the Dp3Tyb model (Fig. 7F, G). We also found no change in human/mouse APP or CTF-β abundance in the Dp(10)2Yey;*App^NL-F/NL-F^* mouse (Fig. 8). We found no evidence of changes to total mouse amyloid-β_40_ or amyloid-β_42_ in young Dp3Tyb compared with wild-type controls (3-months of age) or insoluble human amyloid-β_40_ or amyloid-β_42_ in young (3-months of age) *Dp3Tyb;App^NL-F/NL-F^* compared with *App^NL-F/NL-F^* controls (Fig. 9, 11). These data suggest that the decreased accumulation of human amyloid-β in the Dp3Tyb model is not likely to be the result of changed abundance of APP, CTF-β or amyloid-β in the young brain and a mechanism that decreases aggregation or enhances clearance of amyloid-β may be causal, consistent with the data from the Tc1;*App^NL-F/NL-F^* model.

### In Dp3Tyb mice three-copies of *Bace2* do not raise BACE2 abundance or decrease amyloid-β abundance in the young adult brain

**Figure 10:**
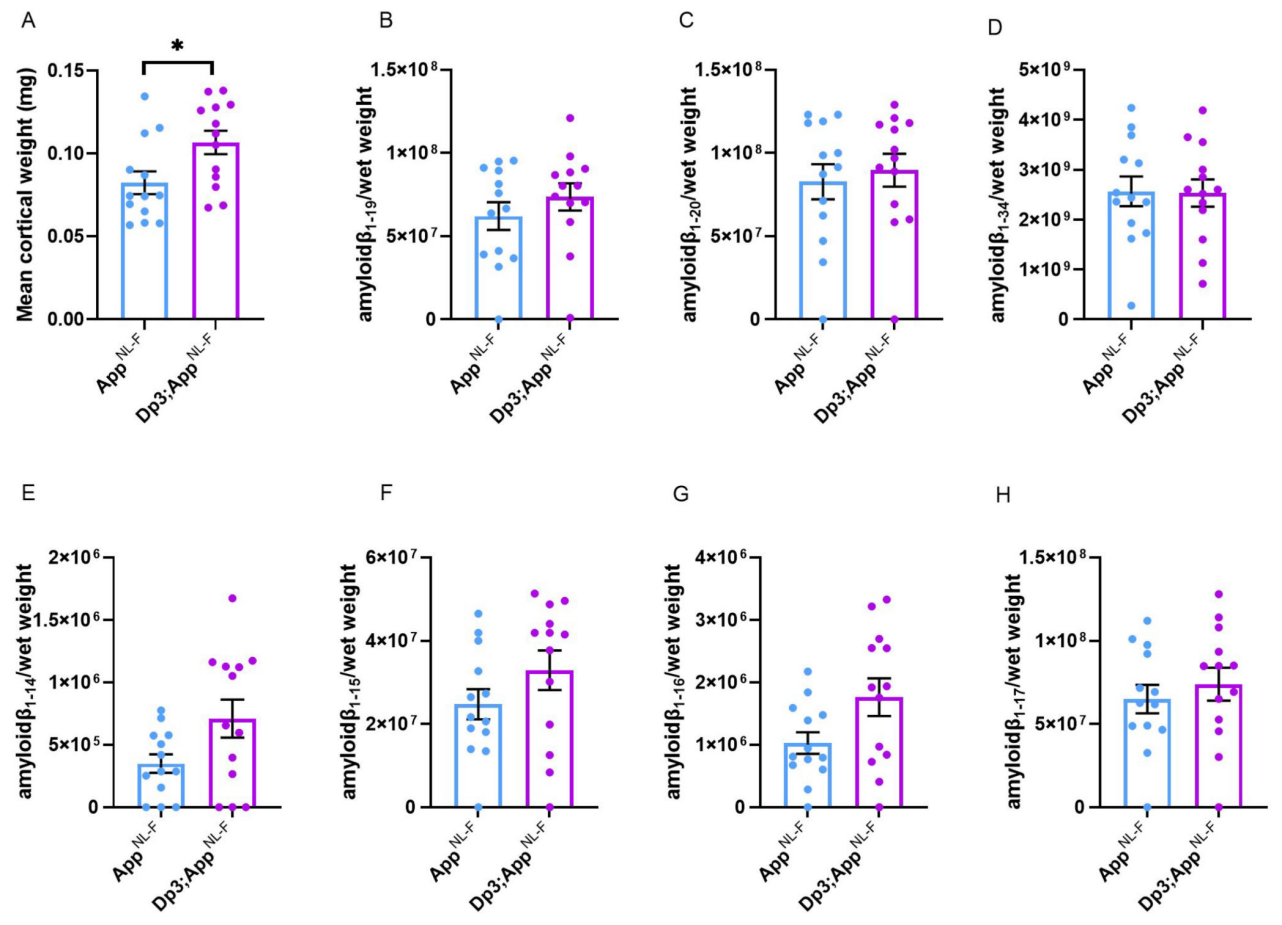
BACE2 amyloid-β cleaved fragments do not have increased abundance in the young cortex of the Dp3Tyb;*App^NL-F/NL-F^* model. LC-MS analysis of immunoprecipated cortical amyloid-β from formic acid fraction normalised to weight of cortical tissue, was used to determine if the Dp3Tyb region was sufficent to alter the abundance of putative BACE2 cleavage products at 3-months of age. **(A)** *Dp3Tyb;App^NL-F/NL-F^* cortex weighs more than *App^NL-F/NL-F^* cortex at 3-months of age (F(1,22) = 7.772, p = 0.011). *App^NL-F/NL-F^* (female n = 8, male n = 5), *Dp3Tyb;App^NL-F/NL-F^* (female n = 4, male n = 9). No significant increase in the abundance of **(B)** amyloid-β_1-19_, (F(1,20) = 0.166 p = 0.688), **(C)** amyloid-β_1-20_ (F(1,20) = 0.274 p = 0.607) or **(D)** amyloid-β_1-34_ (F(1,20) = 0.005 p = 0.942) was observed. No difference in the abundance of **(E)** amyloid-β_1-4_, (F(1,15) = 1.622, p = 0.222), **(F)** amyloid-β_1-15_, (F(1,19) = 0.496, p = 0.490), **(G)** amyloid-β1.16, (F(1,19) = 2.274, p = 0.148) or **(H)** amyloid-β1.17, (F(1,19) = 0.079, p = 0.781) was detected in the cortex of *Dp3Tyb;App^NL-F/NL-F^* compared with *App^NL-F/NL-F^* mice. *App^NL-F/NL-F^* (female n = 8, male n = 5), *Dp3Tyb;App^NL-F/NL-F^* (female n = 4, male = 9). Dp3Tyb abbreviated to Dp3 for clarity. Error bars show SEM, data points are independent mice. For amyloid-β_1-19_ and Amyloid-β_1-20_ n = 1 sample was below the limit of detection per genotype. Amyloid-β_1-4_ n = 3 samples were below the limit of detection per genotype, amyloid-β_1-15_, _1-16_, _1-17_ n = 1 samples were below the limit of detection per genotype. These samples are shown on the graphs but were excluded from ANOVA.

The Dp3Tyb model carries an extra copy of *Bace2*, which encodes a secretase that has been previously reported to cleave APP at the θ site, resulting in the production of amyloid-β_1-19_ (Sun, He et al. 2006, Alic, Goh et al. 2020). BACE2 has also been suggested to clear amyloid-β, leading to reduced accumulation and production of amyloid-β_1-20_ and amyloid-β_1-34_ in an organoid model of DS (Sun, He et al. 2006, Alic, Goh et al. 2020). Thus, we wanted to investigate BACE2 in our AD-DS model. Using western blotting we found that an extra copy of the Dp3Tyb region did not cause BACE2 abundance to be significantly higher in the Dp3Tyb;*App^NL-F/NL-F^* compared with *App^NL-F/NL-F^* cortex (Fig 7A, C), likely because of the underlying high variability in the abundance of this protein in the cortex. We went on to determine if the putative BACE2 amyloid-β degradation products, human-amyloid-β_1-20_ and human-amyloid-β_1-34_ or the APP-θ cleavage product human-amyloid-β_1-19_ were altered by the Dp3Tyb region. No difference in these analytes and human-amyloid-β_1-14_, human-amyloid-β_1-15_, human-amyloid-β_1-16_ or human-amyloid-β_1-17_ was observed between Dp3Tyb;*App^NL-F/NL-F^* and *App^NL-F/NL-F^* cortex at 3-months of age (Fig. 10).

**Figure 11:**
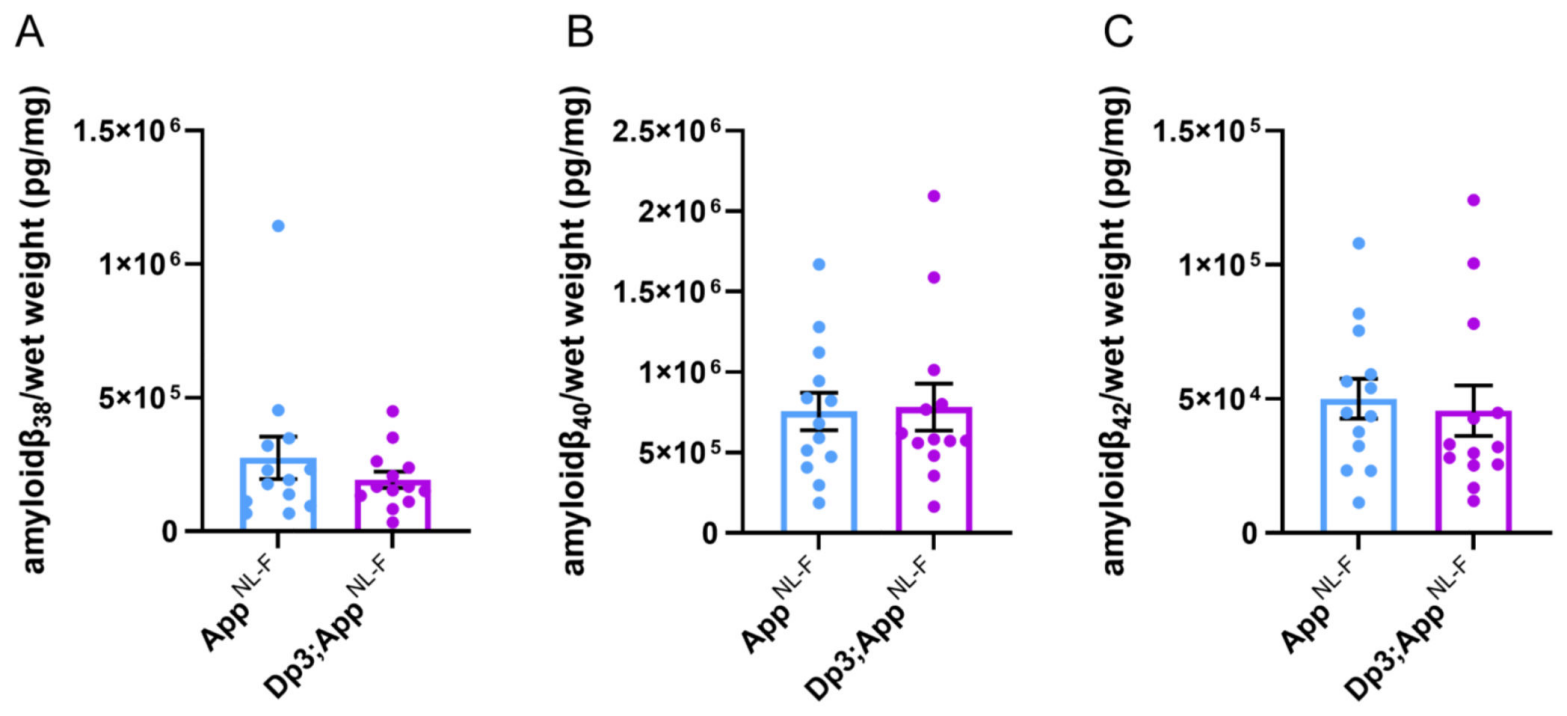
Duplication of the Dp3Tyb region of mouse chromosome 21 orthologous with Hsa21 is not sufficient to decrease amyloid-β biochemical aggregation in the cortex of the *App^NL-F/NL-F^* model at 3-months of age. Total cortical proteins of 3-month-old mice were prepared for mass-spectrometry analysis and amyloid-β abundance analysed in the formic acid soluble fraction by MSD assay (6E10). No difference in the abundance of formic acid soluble **(A)** amyloid-β_38_ (F(1,21) = 1.001, p = 0.328), **(B)** amyloid-β_40_ (F(1,21) = 0.032 p = 0.860) or **(C)** amyloid-β_42_ (F(1,21) = 0.306, p = 0.443) was detected. *App^NL-F/NL-F^* (female n = 8, male n = 5), Dp3Tyb;*App^NL-F/NL-F^* (female n = 4, male n = 9). Dp3Tyb abbreviated to Dp3 for clarity. Error bars show SEM, data points are independent mice.

As we had observed significant variability in the abundance of BACE2 in the cortex, we investigated whether BACE2 protein levels within individual mice predicted the abundance of amyloid-β_1-19_, amyloid-β_1-20_ and amyloid-β_1-34_ in the same animals. No relationships between the level of BACE2 protein and the analytes were observed (amyloid-β_1-19_ R^2^ = 0.0001, amyloid-β_1-20_ R^2^ = 0.0079, amyloid-β_1-34_ R^2^ = 0.0004). These data suggest that differences in BACE2 protein abundance in the young adult mouse brain are not sufficient to cause a detectable alteration in the clearance of amyloid-β or enhanced θ-cleavage. One caveat is that at 3-months of age we cannot yet detect an alteration of the aggregation of human amyloid-β in the Dp3Tyb;*App^NL-F/NL-F^*, as detected by MSD assay after biochemical isolation in formic acid (Fig. 11). Thus, our data could be consistent with a BACE2 aging-dependent mechanism leading to the observed decrease in amyloid-β in the Dp3Tyb;*App^NL-F/NL-F^* model at 8-months of age, but further analysis is required to support this.

## Discussion

Here we use a series of mouse models of DS to identify which combination of Hsa21 genes (other than *APP*) are sufficient to modulate APP/amyloid-β. Our systematic approach identified a region of Hsa21 that contains 38 genes, which is sufficient to decrease the deposition of amyloid-β *in vivo* at an early time-point. This region contains two lead candidate genes *Bace2* and *Dyrk1A*.

DYRK1A is widely expressed in both the developing and adult mouse brain (Marti, Altafaj et al. 2003, Hammerle, Elizalde et al. 2008) and is found in all major cell types in both the human and mouse brain (Zhang, Chen et al. 2014, Zhang, Sloan et al. 2016). It is a primer kinase that can phosphorylate a large number of proteins, including APP, which has been suggested to increase the protein’s abundance (Ryoo, Cho et al. 2008, Branca, Shaw et al. 2017). Inhibition of the kinase in a transgenic mouse model of AD decreased amyloid-β accumulation (Branca, Shaw et al. 2017, Velazquez, Meechoovet et al. 2019). Consistent with this, a decrease in APP and amyloid-β abundance is observed in a mouse model of DS with a normalised *Dyrk1a* gene dose (Garcia-Cerro, Rueda et al. 2017). In the Dp3Tyb model, which contains an additional copy of *Dyrk1a*, we observe no increase in APP abundance (endogenous mouse APP and partially humanised APP_SW_) or evidence of enhanced amyloid-β accumulation. This suggests that in our knock-in model system, 3 copies of *Dyrk1a* are not sufficient to modulate APP protein abundance or promote amyloid-β accumulation. Notably, inhibition of DYRK1A has been suggested as both a possible therapeutic to enhance cognition in people who have DS and as a possible AD therapeutic strategy (De la Torre, De Sola et al. 2014, Yin, Jin et al. 2017, Neumann, Gourdain et al. 2018, Nguyen, Duchon et al. 2018). Our data do not support the use of DYRK1A inhibitors as a treatment strategy to decrease early amyloid-β accumulation in people who have DS. However, inhibiting the kinase may have other beneficial effects, such as to slow or prevent the formation of tau neurofibrillary tangles or to alter the response of cells within the brain to accumulating pathology. Further research is needed, in next generation AD-DS mouse models, to address these key questions.

*BACE2* has been reported to be expressed in a subset of astrocytes and neurons in the human brain (Holler, Webb et al. 2012). In the mouse the gene is expressed in a subset of neurons with the highest level in the CA3 and subiculum and some expression is also reported in oligodendrocytes and astrocytes lining the lateral ventricles (Voytyuk, Mueller et al. 2018). The protein may function as a θ-secretase (decreasing CTF-β), a β-secretase (increasing CTF-β), and as an amyloid-β degrading protease (decreasing amyloid-β) (Sun, He et al. 2006, Abdul-Hay, Sahara et al. 2012); which mechanism predominates in the human brain is not well understood. A previous study in which BACE2 was overexpressed in a wild-type APP over-expressing mouse model reported no evidence of altered amyloid-β_40_ or amyloid-β_42_ abundance in the brain (Azkona, Levannon et al. 2010). Conversely, knocking-down *Bace2* in the *App^NL-G-F^* mouse model of amyloid-β accumulation has been reported to decrease CTF-β and soluble amyloid-β (Wang, Xu et al. 2019). In contrast, reducing *BACE2* copy number in human organoids produced from individuals with DS or *APP*-duplication increased amyloid-β and triggered the formation of deposits within the model system. The authors propose this occurs because of the amyloid clearance function of BACE2, as evidenced by the raised levels of amyloid-β degradation products in trisomic compared to isogenic disomic control (Alic, Goh et al. 2020).

In the Dp3Tyb model that contains an additional copy of *Bace2*, we observe no significant change in CTF-β (endogenous mouse APP and partially humanised APP_SW_), soluble amyloid-β (endogenous mouse and partially humanised APP_SW_) or amyloid-β degradation products in the young adult brain. However, we observe a significant decrease in amyloid-β deposition in older mice (8-months of age) consistent with the role of BACE2 as an amyloid-β degrading protease (Alic, Goh et al. 2020). Further studies are required in aged mice to determine if enhanced amyloid-clearance can be detected, or if BACE2 gene-dose correction is sufficient to reverse the reduction in amyloid accumulation caused by the Dp3Tyb model.

Genes other than *Dyrk1a* or *Bace2* may be responsible for the decreased amyloid-β accumulation observed in our model systems, perhaps via enhancing amyloid-β clearance pathways. However, we previously studied the rate of extracellular clearance of human amyloid-β in an alternative model system (cross of the Tc1 with the J20 *APP* transgenic model) and found no evidence that the amyloid-β clearance rate was modulated by the extra copy of Hsa21 genes (Wiseman, Pulford et al. 2018). In addition, in the same model system we found no evidence of increased abundance of the key clearance enzymes neprilysin or insulin degrading enzyme in the young adult brain. Changes to intracellular clearance/formation of amyloid-β may contribute to the alterations in accumulation of the peptide reported here. Notably DS is associated with significant alterations to endosomal biology (Botte and Potier 2020); neuronal endosomes have a key role in amyloid-β formation and astrocyte and microglia endosomes in amyloid-β clearance. Further studies focussing on cell type specific effects of extra chromosome 21 genes are required to understand this complex and important biology.

DS is caused by the increase in the abundance of Hsa21 gene products; however, not all genes on the chromosome are dosage-sensitive in all contexts. For example, *APP* dosage sensitivity has been reported to vary over life-span (Choi, Berger et al. 2009) and BACE2 has been reported to be both dosage-sensitive (Barbiero, Benussi et al. 2003) and insensitive in the human brain (Cheon, Dierssen et al. 2008, Holler, Webb et al. 2012). In contrast, the abundance of DYRK1A is highly sensitive to gene dose in the vast majority of reported studies (Arbones, Thomazeau et al. 2019), including previously in the adult brain of the Tc1 mouse model (Sheppard, Plattner et al. 2012, Ahmed, Dhanasekaran et al. 2013, Naert, Ferre et al. 2018). Consistent with this, here we report a significant increase in DYRK1A in the adult brain of the new Dp3Tyb model. However we could not detect a significant increase in BACE2, this lack of statistical significance is likely caused by the higher intra-animal variation in abundance of this protein in the mouse brain. Similar overlapping levels of *Bace2* gene expression have previously been reported at RNA level in the brain of an alternative mouse model of DS compared with euploid controls (Sultan, Piccini et al. 2007).

The relative abundance of proteins in AD-DS model systems is particularly important, as Down syndrome is caused by an inbalance in the relative abundance of gene products. Previous research using *APP* transgenic models, which over express APP protein, may mask the subtle but physiologically relevant effects of the 50% increase in the abundance of other Hsa21 gene products. This study addresses this limitation, here we systematically investigate the effect of additional copies of Hsa21 gene orthologues, other than *App*, on APP biology in a knock-in mouse model system. However, because of technical limitations we were not able to study the effect of an extra copy of genes located close to *App*, including the role of key genes such as *Adamts1* (Kunkle, Grenier-Boley et al. 2019) and *Usp25* (Zheng, Li et al. 2021). We were also unable to determine if genes located far apart on the chromosome act synergistically to cause a modulation of APP biology. Thus our data does not preclude a multigenic role for the genes in the Dp(10)2Yey or Dp(17)3Yey regions when combined with other Hsa21 genes. A further limitation is the use of AD-associated mutations in APP to drive pathology as these mutations alter the subcellular localisation and processing of APP (Sasaguri, Nilsson et al. 2017), which may modulate the effect of trisomy of Hsa21 on these processes. We have partially addressed this issue by undertaking a side-by-side comparison of mouse APP/amyloid-β and partially humanised APP/amyloid-β.

We studied two time-points in our model systems. Firstly, the young adult brain at 3 months of age, prior to the accumulation of human amyloid-β in the *App^NL-F^* in order to understand the modulation of early upstream processes by the additional copies of Hsa21 orthologues. Secondly the middle-aged adult brain at 8-months of age, which in the *App^NL-F^* mouse, models the initiation of amyloid-β accumulation (Saito, Matsuba et al. 2014). Thus in this study we focus on how additional copies of Hsa21 gene orthologues modulate the earliest stages of AD pathology; the formation and early accumulation of amyloid-β in the brain. We note that the effect of the additional genes may differ later in disease, including during the maturation of amyloid-β plaques, the formation of tau neurofibrillary tangles and the response of astrocytes, microglia and neurons to AD pathology. We studied both male and female mice, to ensure the generalisability of our findings, as sex is know to modulate APP/amyloid-β in mouse models (Jankowsky and Zheng 2017). We did not find evidence that sex modulated the effect of additional Hsa21 genes on amyloid accumulation in either the Tc1 or Dp3 models.

Our results indicate that an additional copy of genes on Hsa21 in people who have DS can modulate key AD biology. The extent of this effect is likely to differ between individuals both because of genetic variation on Hsa21 and differences in life-style factors that modulate dementia risk. This may contribute to the variation in age of onset of both pathology and dementia observed in people who have DS.

## Conclusion

DS is a complex condition that alters multiple aspects of neurobiology and physiology. Here we demonstrate using physiological mouse models that an additional copy of Hsa21 genes reduces the accumulation of amyloid-β within the brain; one of the earliest steps in the AD pathogenic process. Thus trisomy of Hsa21 may partially protect individuals who have DS from the accumulation of amyloid-β, resultant from their extra copy of *APP*. Moreover we predict that individuals who have DS will accumulate amyloid-β more slowly than other groups who develop other genetic forms of early-onset AD, including that caused by duplication of *APP*. However, multiple studies of adults who have DS demonstrate that these individuals do accumulate substantial amyloid-β within their brains by mid-life, thus the effect of an additional copy of other genes on the chromosome is not sufficient to delay pathology development and AD primary prevention therapies targeting amyloid-β are likely to significantly benefit individuals who have DS (Fortea, Zaman et al. 2021). Treatments for AD-DS and other medical conditions associated with DS must take into account the differences in biology of individuals with the syndrome; to ensure that interventions do not reverse the beneficial effect of the additional genes.

## Figures and Tables

**Table 1:** Summary of Mouse Cohorts Summary of the cohorts of mice, the cross(es) used to generate them, the age at which the cohort was sacrificed, the outcome measured and the figure in which the data are presented.

Cohort	Crosses	Age	Outcomes	Figure	Female ‘n’				Male ‘n’			
					WT	Tc1/Dpx	*App* ^*NL-F/NL-F*^	Tc1/Dpx; *App*^*NL-F/NL-F*^	WT	Tc1/Dpx	*App* ^*NL-F/NL-F*^	Tc1/Dpx; *App*^*NL-F/NL-F*^
1	Tc1;*App*^NL-F/+^ and *App^NL-F/+^*	3-months	APP and APP-CTF abundance	Figure 3	4	3	2	4	4	5	2	4
2	Dp(10)2Yey;*App^NL-F/+^* and App^NL-F/+^	3-months	APP and APP-CTF abundance	Figure 8	1	0	0	2	4	8	9	4
3	Dp3Tyb-*App^NL-F/+^* and *App^NL-F/+^* Dp3Tyb and C57BL/6J *App^NL-F/+^* and *App^NL-F/+^*	3-months	APP, APP-CTF, DYRK1A and BACE2 abundance Human amyloid-β fragments (only *App^NLF^* carriers analysed)	Figure 7 Figure 10 & 11	6	5	8	5	11	7	7	8
4	Dp3Tyb and C57BL/6J	3-months	Mouse amyloid-β abundance	Figure 9	5	7	N/A	N/A	11	12	N/A	N/A
5	Tc1*;App^NL-F/+^* and *App^NL-F/+^*	8-months	82E1 plaque counts Human amyloid-β abundance	Figure 2	5	5	15	8	0	3	8	10
6	Dp3Tyb-*App^NLF/+^* and *App^NL-F/+^ App^NL-F/+^* and *App^NL +^*	8-months	82E1 plaque counts Human amyloid-β abundance	Figure 4 Figure 5 & 6	2	Not available because of breed scheme	2	9	2	Not available because of breed scheme	7	5
7	Dp(10)2Yey;*App^NL-F/+^* and *App^NL-F/+^*	8-months	82E1 plaque counts Human amyloid-β abundance	Figure 4 Figure 5 & 6	2	2	4	6	4	1	5	3
8	Dp(17)3Yey; *App^NL-F/+^* and *App^NL-F/+^*	8-months	82E1 plaque counts Human amyloid-β abundance	Figure 4 Figure 5 & 6	3	3	7	7	3	3	5	7

**Table 2:** Summary of negative control samples, which do not carry an *App^NL-F^* allele and do not produce human amyloid-β, used in MSD assays. Negative control samples from mice that do not carry the *App^NL-F^* allele and do not produce human amyloid-β were assayed by 6E10 MSD assay alongside samples homozygous for the *App^NL-F^* allele and which do produce human amyloid-β. The epitope for 6E10 lies within the region of sequence difference between human and mouse Aβ, the antibody is against the human sequence. In some cases as indicated all samples without an *App^NL-F^* allele were below the limit of detection for the assay and thus mean and standard deviations are not available (N/A)

Figure	Measurement	Genotype	Total ‘n’	Below the limit of detection	Above Limit of Detection		
					n	Mean (pg/mg)	Standard deviation
Figure 5A	Gnd HCl amyloid-β_42_/ total brain weight	WT	4	4	0	N/A	N/A
Figure 5A	Gnd HCl amyloid-β_42_/ total brain weight	Dp3Tyb	N/A	N/A	N/A	N/A	N/A
Figure 5B	Gnd HCl amyloid-β_42_/total brain weight	WT	6	0	6	0.505	0.444
Figure 5B	Gnd HCl amyloid-β_42_/ total brain weight	Dp(10)2Tyb	3	0	3	0.59	0.411
Figure 5C	Gnd HCl amyloid-β_42_/ total brain weight	WT	5	4	1	6.154	N/A
Figure 5C	Gnd HCl amyloid-β_42_/ total brain weight	Dp(17)3Tyb	5	5	0	N/A	N/A
Figure 5D	1% Triton amyloid-β_42_/total brain weight	WT	4	3	1	0.002	N/A
Figure 5D	1% Triton amyloid-β_42_/ total brain weight	Dp3Tyb	Not available	N/A	N/A	N/A	N/A
Figure 5E	1% Triton amyloid-β_42_/ total brain weight	WT	6	5	1	0.009	N/A
Figure 5E	1% Triton amyloid-β_42_/ total brain weight	Dp(10)2Tyb	3	2	1	0.007	N/A
Figure 5F	1% Triton amyloid-β_42_/ total brain weight	WT	5	2	3	0.475	0.814
Figure 5F	1% Triton amyloid-β_42_/ total brain weight	Dp(17)3Tyb	5	3	2	0.074	N/A
Figure 5G	Tris amyloid-β_42_/ total brain weight	WT	4	3	1	0.015	N/A
Figure 5G	Tris amyloid-β_42_/ total brain weight	Dp3Tyb	Not available	N/A	N/A	N/A	N/A
Figure 5H	Tris amyloid-β_42_/total brain weight	WT	6	5	1	0.006	N/A
Figure 5H	Tris amyloid-β_42_/ total brain weight	Dp(10)2Tyb	3	2	1	0.007	N/A
Figure 5I	Tris amyloid-β_42_/ total brain weight	WT	5	2	3	0.304	0.517
Figure 5I	Tris amyloid-β_42_/ total brain weight	Dp(17)3Tyb	5	2	3	0.011	0.009
Figure 6A	Gnd HCl amyloid-β_40_/total brain weight	WT	6	0	6	2.097	1.246
Figure 6A	Gnd HCl amyloid-β_40_/total brain weight	Dp(10)2Tyb	3	0	3	2.255	1.796
Figure 6B	Gnd HCl amyloid-β_40_/ total brain weight	WT	5	5	0	N/A	N/A
Figure 6B	Gnd HCl amyloid-β_40_/ total brain weight	Dp(17)3Tyb	5	5	0	N/A	N/A
Figure 6C	1% Triton amyloid-β_42_/ total brain weight	WT	6	4	2	0.058	0.004
Figure 6C	1% Triton amyloid-β_42_/ total brain weight	Dp(10)2Tyb	3	1	2	0.035	0.023
Figure 6D	1% Triton amyloid-β_42_/ total brain weight	WT	5	4	1	3.241	N/A
Figure 6D	1% Triton amyloid-β_42_/ total brain weight	Dp(17)3Tyb	5	5	5	N/A	N/A

## Significance Statement

Trisomy of chromosome 21 is a commonly occurring genetic risk factor for early-onset Alzheimer’s disease, which has been previously attributed to people with Down syndrome having three copies of the *APP* gene, which is encoded on chromosome 21. However, we have shown that an extra copy of other chromosome 21 genes modifies AD-like phenotypes *independently* of *APP* copy number (Wiseman et al. 2018, Brain; Tosh et al. 2021 Scientific Reports). Here, we use a mapping approach to narrow-down the genetic cause of the modulation of pathology; demonstrating that gene(s) on chromosome 21 decrease amyloid-β accumulation in the brain, independently of alterations to full-length APP or C-terminal fragment abundance and that just 38 genes are sufficient to cause this.

## Abbreviations

AD: Alzheimer’s disease
AD-DS: Alzheimer’s disease in Down syndrome
APP: Amyloid precursor protein
BSA: Bovine serum albumin
DS: Down syndrome
FA: Formic acid
IP: Immunoprecipitation
LC-MS: Liquid chromatography-mass spectrometry
MS: Mass spectrometry
MSD: Meso Scale Discovery
TBS: Phosphate-buffered saline (PBS) Standard error of the mean (SEM) Tris-buffered saline
